# Trends in food and beverage purchases in informal, mixed, and formal food outlets in Mexico: ENIGH 1994–2020

**DOI:** 10.3389/fpubh.2023.1151916

**Published:** 2023-05-24

**Authors:** Ana Paula Domínguez-Barreto, Irene Farah, Nancy López-Olmedo, Carolina Perez-Ferrer, Yenisei Ramírez-Toscano, Tonatiuh Barrientos-Gutiérrez, Dalia Stern

**Affiliations:** ^1^Center for Research on Population Health, National Institute of Public Health, Cuernavaca, Morelos, Mexico; ^2^Department of City and Regional Planning, University of California, Berkeley, Berkeley, CA, United States; ^3^CONACyT–Center for Research on Nutrition and Health, National Institute of Public Health, Cuernavaca, Morelos, Mexico; ^4^CONACyT–Center for Research on Population Health, National Institute of Public Health, Cuernavaca, Morelos, Mexico

**Keywords:** food purchases, retail food environment, food outlets, households, informal food outlets

## Abstract

**Background:**

The retail food environment in Mexico is characterized by the co-existence of both, formal and informal food outlets. Yet, the contribution of these outlets to food purchases over time has not been documented. Understanding the longitudinal trends where Mexican households purchase their foods is critical for the development of future food retail policies.

**Methods:**

We used data from Mexico’s National Income and Expenditure Survey from 1994 to 2020. We categorized food outlets as formal (supermarkets, chain convenience stores, restaurants), informal (street markets, street vendors, acquaintances), and mixed (fiscally regulated or not. i.e., small neighborhood stores, specialty stores, public markets). We calculated the proportion of food and beverage purchases by food outlet for each survey for the overall sample and stratified by education level and urbanicity.

**Results:**

In 1994, the highest proportion of food purchases was from mixed outlets, represented by specialty and small neighborhood stores (53.7%), and public markets (15.9%), followed by informal outlets (street vendors and street markets) with 12.3%, and formal outlets from which supermarkets accounted for 9.6%. Over time, specialty and small neighborhood stores increased 4.7 percentage points (p.p.), while public markets decreased 7.5 p.p. Street vendors and street markets decreased 1.6 p.p., and increased 0.5 p.p. for supermarkets. Convenience stores contributed 0.5% at baseline and increased to 1.3% by 2020. Purchases at specialty stores mostly increased in higher socioeconomic levels (13.2 p.p.) and metropolitan cities (8.7 p.p.) while public markets decreased the most in rural households and lower socioeconomic levels (6.0 p.p. & 5.3 p.p.). Supermarkets and chain convenience stores increased the most in rural localities and small cities.

**Conclusion:**

In conclusion, we observed an increase in food purchases from the formal sector, nonetheless, the mixed sector remains the predominant food source in Mexico, especially small-neighborhood stores. This is concerning, since these outlets are mostly supplied by food industries. Further, the decrease in purchases from public markets could imply a reduction in the consumption of fresh produce. In order to develop retail food environment policies in Mexico, the historical and predominant role of the mixed sector in food purchases needs to be acknowledged.

## 1. Introduction

The retail food environment is an important determinant of population nutrition and nutrition-related chronic diseases, including obesity (1–4). Retail food environments comprehend the availability, affordability, and quality of foods and beverages. Such factors influence the food choices of individuals by making the purchase of certain foods more convenient (5). Yet, interventions targeting the retail food environment remain scarce (6), and most of available literature seeking to understand the retail food environment has focused on high-income countries (HIC). Thus, understanding local household food purchasing preferences in low- and middle-income countries (LMIC) is key to identify the outlets where most people shop for food (2, 5, 7, 8).

In a cross-sectional analysis in 2018, Mexican households purchased most of their foods and beverages in outlets of the mixed sector, which are traditional small outlets that can be fiscally regulated or not (9, 10). Up to 70% of the food and beverage purchases were done in the mixed sector, including purchases at small neighborhood stores, specialty stores, public markets, and low-budget restaurants. In contrast, only 15% of purchases were made in the formal sector (i.e., supermarkets, chain convenience stores, restaurants), 14% corresponded to the informal sector (street vendors, street markets and acquaintances), and 1% to other (9). This purchase pattern diverges from purchases in HIC, which are concentrated in the formal sector, but it is similar to what has been observed in other LMICs (2, 3). While these analyses were informative of the status of food purchases in 2018, they did not provide information about changes in purchases over time. Understanding the foods and beverages purchasing trends could shed light into the contribution of the mixed, formal, and informal sectors to the populations health and nutrition status over time.

Extensive literature has documented how the number of formal food outlets, especially supermarkets and chain convenience stores, has increased over time in LMIC (11–16). During the 1990s, the trade liberalization led to the “modernization” of local markets which resulted in a rapid growth of supermarkets and chain convenience stores in LMIC, affecting the supply, demand, and the number of small traditional stores (10, 13–15). However, in Mexico, this trend is slowing down, and while the relative increase in the number of supermarkets and chain convenience stores is high, traditional stores (mixed food outlets) continue to be the main source of food and beverages (15). Nonetheless, the market “modernization” led to an increased demand for non-staples and ultra-processed foods, making small traditional stores an important source of ultra-processed foods as they started to be supplied by the food industries (3, 17). While this has provided a clear picture of the recent trends in the number of food outlets from the formal and mixed sector, longitudinal information on where households’ shop for food in general and across socioeconomic and urbanicity strata remains unknown.

The description of the contribution of mixed, formal and informal outlets to food purchases in Mexico over time is key to identify which food outlets are the best target for policies that aim to regulate and improve the retail food environment. Moreover, understanding these trends is important because it can establish the limits of the regulatory capacity of the retail food environment, especially for the informal and mixed sectors. To address these gaps, we estimated the trends of food and beverage purchases in outlets of the mixed, formal, and informal food sectors from 1994 to 2020 using Mexico’s National Income and Expenditure Surveys (ENIGH) and evaluated whether these trends differed across socioeconomic and urbanicity strata.

## 2. Methods

### 2.1. Data sources

We used 14 cross-sectional surveys of the National Income and Expenditure Survey (Encuesta Nacional de Ingresos y Gastos de los Hogares, ENIGH) from 1994 to 2020 conducted by the National Institute of Statistics and Geography of Mexico. All ENIGH surveys, except for 1994, were collected biennially, between August and November. The ENIGH is a probabilistic survey with a two-stage stratified clustered sampling design, representative at a national level (18). Starting in 2016, surveys also became representative at the state level. ENIGH uses the household as the study unit and collects information on income as well as daily expenses. Additionally, ENIGH collects information on household’s sociodemographic characteristics and city size (18).

Food and beverage expenditure was collected by surveying households daily for seven consecutive days. Household food and beverage expenses were reported by the household member responsible for the purchases and complemented by a food diary kept by each household member. The food diary contains information on the name of foods and beverages purchased, quantity purchased (liters or kilograms), the price paid (Mexican pesos), and the type of food outlet where purchases were made (19).

Outlet categories in ENIGH varied across time, thus, to analyze different food outlets within the informal, mixed and formal sectors, we used two samples covering different time periods for this study. The first, included data from 1994 to 2020, providing a greater temporary coverage of food purchasing trends. Starting in 2006, ENIGH collected more disaggregated data for outlet types that were previously collected as a single outlet. Thus, the second sample included information from 2006 to 2020. This second analysis was conducted to provide a deeper understanding of the individual outlets from the informal and mixed sector. From 1994 to 2020, ENIGH included a total of 428,122 households, varying from 12,815 households in 1994 to 89,006 in 2020. We excluded households that did not report any food or beverage purchases (*n* = 4,232, 0.99%), resulting in an analytical sample of 423,890 households. For the analyses of 2006 to 2020, the analytical sample included 340,443 households.

### 2.2. Food and beverage outlets

Surveys conducted between 1994 and 2000 included 12 outlet categories, while surveys collected between 2010 and 2020 included 18 categories (Supplementary Table 1). We created two outlet classifications (Table 1). The first classification (1994 to 2020) included nine categories: (1) street markets (*tianguis*) and street vendors; (2) acquaintances (starting in 2010); (3) public markets; (4) specialty stores and small neighborhood stores (*abarrotes*); (5) low-budget restaurants; (6) restaurants, cafes, bars; (7) supermarkets and department stores; (8) chain convenience stores (starting in 2006); and (9) others (outlets included in “others” are described in Table 1). We used the second food outlet classification to look at specialty stores and small neighborhood stores separately, as well as street markets and street vendors. In this classification we also analyzed supermarkets as a sole category (excluding department stores). The second classification (2006 to 2020) included 11 categories: (1) street markets (*tianguis*); (2) street vendors; (3) acquaintances; (4) public markets; (5) specialty stores; (6) small neighborhood stores (*abarrotes*); (7) low-budget restaurants; (8) restaurants, cafes, bars; (9) supermarkets; (10) chain convenience stores; and (11) others. We categorized street markets, street vendors, and acquaintances in the informal sector, defined as unregistered establishments under tax or social security laws (20). Public markets, specialty stores, small neighborhood stores, and low-budget restaurants can either belong to the formal or informal categories, since these outlets are small, they tend to be family-owned, and could be fiscally unregulated. Yet, ENIGH does not provide the necessary information to classify these types of outlets by fiscal or labor regimes. Therefore, we classified these outlets as mixed. Finally, we classified as formal outlets those that are most likely fiscally regulated establishments such as restaurants, cafes, bars, supermarkets, and chain convenience stores.

**Table 1:** Food and beverage outlet classifications and their characteristics (18).

1994–2020 classification	2006–2020 classification	Characteristics	Fiscal regime
Street markets (*tianguis*) and street vendors	Street markets (*tianguis*)	Set of vendors who are not fixed and set up in a certain time during the day to market their products in stands.	Informal
	Street vendors	Stands in public roads or spaces, vendors from home to home, and vehicles that offer goods or services (mobile vendors) are considered street vendors.	Informal
Acquaintances	Acquaintances	People dedicated to the sale of products and food that do not have a fixed establishment. They sell foods to neighbors, friends, family, or workplaces.	Informal
Public markets	Public markets	Public space where retail sales take place in different fixed establishments	Mixed
Specialty and small neighborhood stores (*abarrotes*)	Specialty Stores	Outlets that are dedicated to the commercialization of a single product or service: chicken shops, tortilla shops, butcher shops, among others.	Mixed
	Small neighborhood stores (*abarrotes*)	Outlets dedicated to the retail sale of various products.	Mixed
Low-budget restaurants	Low-budget restaurants	Small establishments that sell prepared foods and offer low-budget, affordable meals and the selection of foods is restricted to specific meals. (e.g., *fonda*, *cocina económica*, *lonchería*, *taquería*)	Mixed
Restaurants, cafes, bars	Restaurants, cafes, bars	Public establishments that sell prepared foods and beverages, and are consumed *in situ*, they offer alcoholic beverages, accept credit cards, and offer a menu.	Formal
Supermarkets and department stores	Supermarkets	Large commercial stores, divided into specialized departments, by items or products and have self-service for the public. They are distinguished by the sale of fresh and canned products.	Formal
		Department stores are big establishments with specialized departments. These usually exclude the sale of fresh or perishable foods (e.g., *Liverpool, Sears, El Palacio de Hierro*).	
Chain convenience stores	Chain convenience stores	Commercial chains that sell food products, packaged snacks and cookies, soft drinks, bottled water, alcoholic beverages, among others. These outlets are less than 500 m^2^, with >18 business hours, and open 365 days a year. (e.g., *7-eleven*, *Oxxo*).	Formal
Others	Others	Wholesalers, department stores (in the 2006–2020 classification), international purchases, government establishments that provide food, and internet purchases (included from 2010 onwards).	Other

### 2.3. Urbanicity and education level of the head of the household

ENIGH classifies the localities’ urbanicity according to the number of inhabitants. Rural localities are those with less than 2,500 inhabitants, small cities with 2,500 to 14,999 inhabitants, medium cities with 15,000 to 99,999 inhabitants, and metropolitan cities with more than 100,000 inhabitants (19). The definition of income in ENIGH has varied over time. Thus, we used the highest level of completed education of the head of the household as a proxy for socioeconomic status. ENIGH considers nine categories of education, that we grouped into 4 mutually exclusive categories: (1) without formal education, (2) primary school, (3) high school, and (4) higher education.

### 2.4. Statistical analysis

For each survey, we calculated the contribution of the households’ food and beverage expenses by food outlet type to the total food and beverages expenses (percent expenditure by outlet type. From here onwards, we refer to this as food purchases). Households that did not report food or beverage purchases in a specific outlet were included in the analysis with a percentage contribution of zero. Additionally, for each survey, we estimated the contribution of each outlet type to total household food and beverage purchases stratified by urbanicity and education level. All analyses were conducted in Stata 16 using the SVY command to account for the complex survey design and weighed to generate nationally representative estimates. Weights were created for every ENIGH survey to account for the selection probabilities and survey non-response to match the estimated population for every survey year from the National Institute of Statistics and Geography (21).

## 3. Results

Table 2 shows the sociodemographic characteristics of the ENIGH sample over time among households reporting expenditures on food and beverages. The proportion of households in the lowest education levels decreased over time, while households with the highest educational levels increased. For example, in 1994, 16.9% of head of households reported having no formal education. By 2020, this proportion decreased to 6.3%. In contrast, households with higher education increased from 7.3 to 14.1% from 1994 to 2020. Over time, the distribution of households by urbanicity remained stable, with 50% of the households located in metropolitan cities.

**Table 2:** Sociodemographic characteristics of households by year: the National Income and Expenditure Survey, 1994–2020.

Year	1994	1996	1998	2000	2002	2004	2006
Total households (n)	12,527	13,795	10,670	9,929	16,914	22,276	20,608
Education level^a^, %							
Without formal education	16.9	13.6	12.8	12.4	13.5	11.1	9.8
Primary school	49.6	49.7	49.5	46.5	45.0	46.2	56.8
High school	24.4	27.3	27.8	29.4	31.5	32.2	22.3
Higher education	7.3	7.2	7.8	10.0	8.6	10.5	11.1
Urbanicity, %							
Rural localities	23.2	22.2	22.3	22.4	23.3	22.3	22.1
Small cities	13.4	13.5	13.4	13.4	13.4	13.7	13.1
Medium cities	14.3	13.4	13.3	13.4	13.4	13.9	14.7
Metropolitan cities	49.1	50.9	51.0	50.9	49.9	50.1	50.1
Year	2008	2010	2012	2014	2016	2018	2020
Total households (n)	29,217	27,415	8,924	19,355	69,850	74,194	88,216
Education level^a^, %							
Without formal education	9.3	8.9	8.9	7.7	7.1	6.7	6.3
Primary school	44.6	42.4	39.9	38.0	36.2	34.5	33.6
High school	35.1	36.7	39.8	41.5	43.6	45.0	46.0
Higher education	11.0	11.9	11.4	12.8	13.1	13.9	14.1
Urbanicity, %							
Rural localities	21.3	21.3	21.9	21.9	21.7	23.0	21.5
Small cities	13.8	13.8	13.3	13.5	13.9	14.0	13.7
Medium cities	14.5	14.4	14.4	14.8	14.5	14.7	14.8
Metropolitan cities	50.3	50.5	50.5	49.8	49.9	48.2	50.0

a Completed education level of the head of the household.

### 3.1. Trends in food and beverage purchases (% expenditure) in the informal, mixed and formal sectors from 1994 to 2020

**Figure 1:**
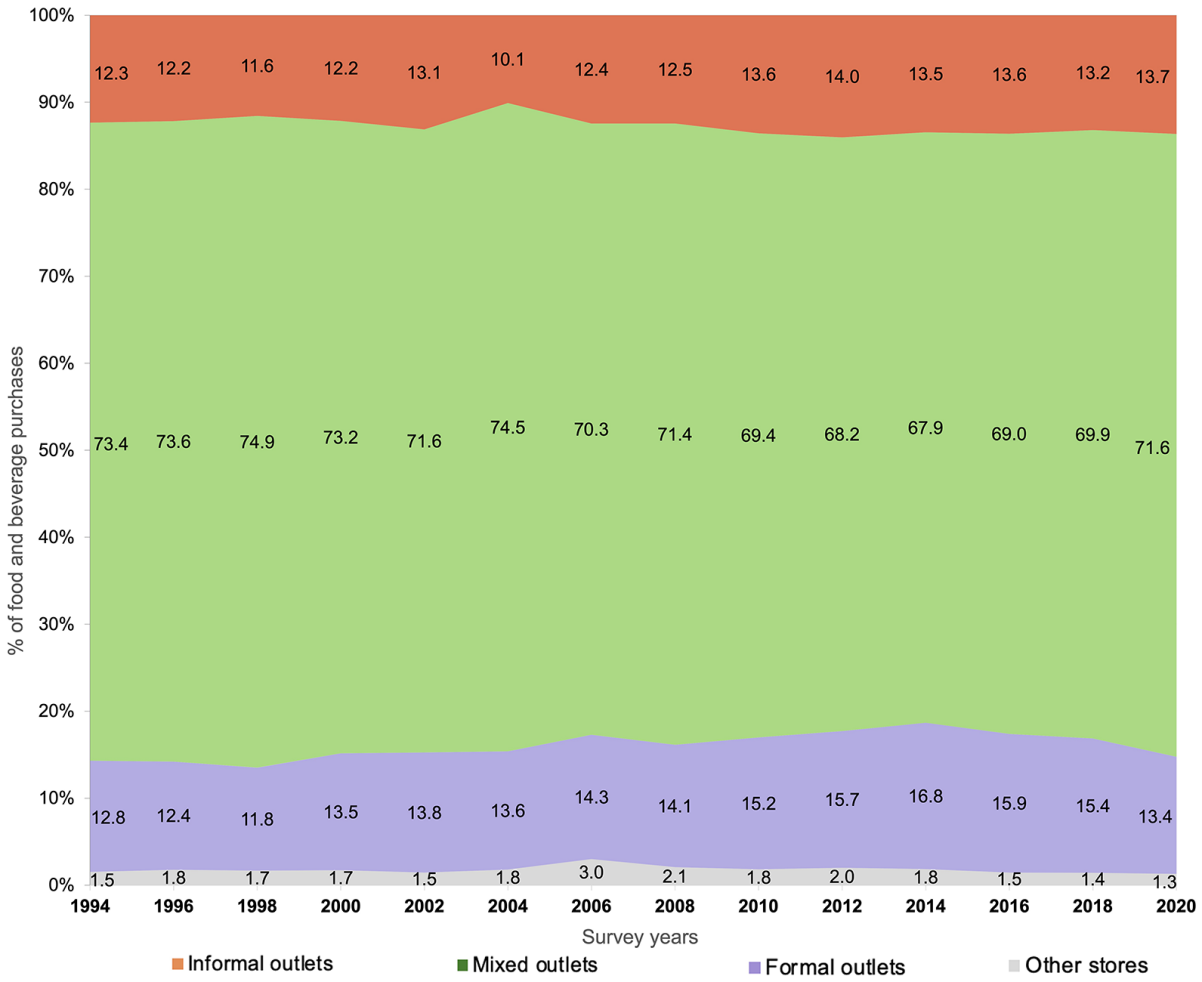
Trends in food purchases (% expenses) at the informal, mixed and formal sector: ENIGH 1994–2020. *Informal* outlets include street markets, street vendors and acquaintances (orange); *mixed* outlets include public markets, low-budget restaurants, and specialty stores (green); *formal* outlets include supermarkets, department stores, restaurants, cafes, bars, and chain convenience stores (purple).

At baseline, the highest proportion of food purchases was represented by mixed outlets (73.4%), followed by formal (12.8%) and informal outlets (12.3%). By 2020, mixed outlets decreased 1.8 percentage points (p.p.), (95% CI −2.6, −0.9), formal outlets increased 2.7 p.p. (95% CI 2.0, 3.3) from 1994 to 2018 and decreased 2.0 p.p. (95% CI −2.4, −1.7) by 2020. Food purchases in informal outlets increased 1.3 p.p. (95% CI 0.7, 1.9) over time (Figure 1).

### 3.2. Trends in food and beverage purchases (% expenditure) by outlet type from 1994 to 2020

**Figure 2:**
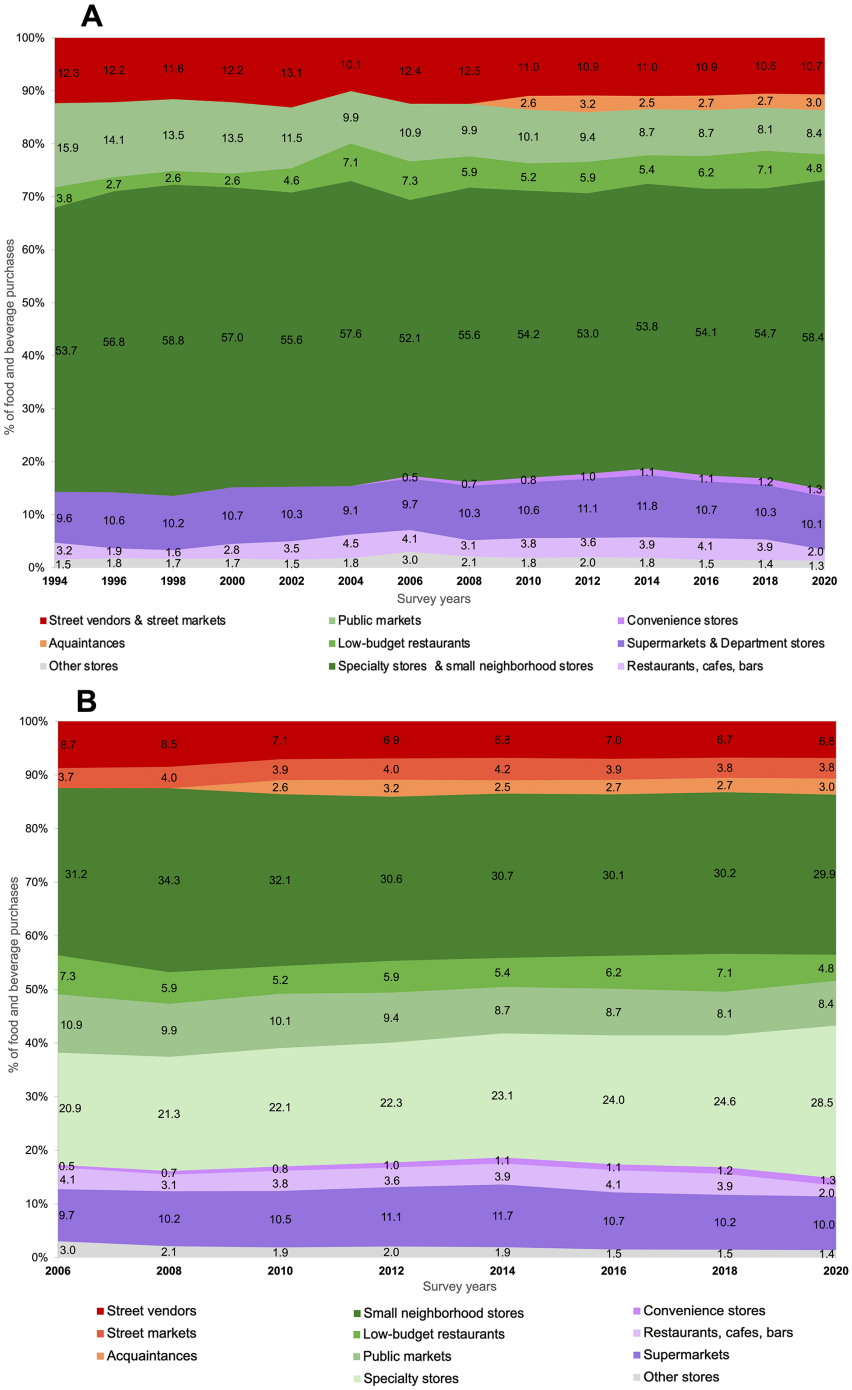
Trends in food purchases (% expenses) by outlet type. **(A)** ENIGH 1994–2020, **(B)** ENIGH 2006–2020. *Informal* outlets include street markets, street vendors, and acquaintances (red and orange); *mixed* outlets include small neighborhood stores, public markets, low-budget restaurants, and specialty stores (green); *formal* outlets include supermarkets, restaurants, cafes, bars, and chain convenience stores (purple).

Figure 2A shows the trends of food and beverage purchases by outlet type from 1994 to 2020. In 1994, the highest proportion of food purchases was represented by mixed outlets. Particularly, specialty stores and small neighborhood stores (53.7%), followed by public markets (15.9%). Over time, the contribution of specialty stores and small neighborhood stores to total food and beverage purchases increased 4.7 p.p. (95% CI 3.8, 5.7), while public markets decreased 7.5 p.p. (95% CI −8.2, −6.9). Purchases in low-budget restaurants increased 3.3 p.p. (95% CI 2.9, 3.7) between 1994 and 2018 and decreased 2.3 p.p. (95% CI −2.5, −2.0) in 2020. At baseline, purchases from informal outlets were mostly represented by street vendors and street markets (12.3%), and decreased 1.7 p.p. (95% CI −2.3, −1.1) over time. Food purchases from acquaintances represented 2.6% of the total purchases in 2010 and remained stable over time. The highest proportion on food purchases in formal outlets in 1994 was from supermarkets and department stores (9.6%), followed by restaurants, cafes and bars (3.2%). Over time, food purchases from supermarkets and department stores remained stable (+0.5 p.p., 95% CI 0.0, 1.1). Food purchases from restaurants, cafes, and bars increased 0.7 p.p. (95% CI 0.4, 1.1) between 1994 and 2018 and decreased 1.9 p.p. (95% CI −2.0, −1.7) in 2020. Convenience stores contributed 0.5% at baseline (2006) and increased to 1.3% by the end of the period.

The outlet categorization from 2006 to 2020 allowed us to study household purchases from small neighborhood stores (abarrotes) and specialty stores separately. The largest contribution to total food and beverage purchases in 2006 was from small neighborhood stores (31.2%), followed by specialty stores (20.9%). Food purchases from specialty stores increased 7.6 p.p. (95% CI 7.0, 8.2), but decreased in small neighborhood stores by 1.3 p.p. (95% CI −2.00, −0.6). This categorization also allowed us to study household purchases from street markets and street vendors separately. We found that street vendors accounted for most of the food purchases from the informal sector in 2006 (8.7%), while street markets represented 3.7%. Food purchases from street vendors decreased 1.9 p.p. (95% CI −2.3, −1.5), while purchases from street markets remained stable. In this categorization, supermarkets were studied excluding department stores. However, trends were similar to those described earlier (Figure 2B).

### 3.3. Trends in food and beverage purchases (% expenditure) by outlet type stratified by education level of the head of the household and urbanicity from 2006 to 2020

**Figure 3:**
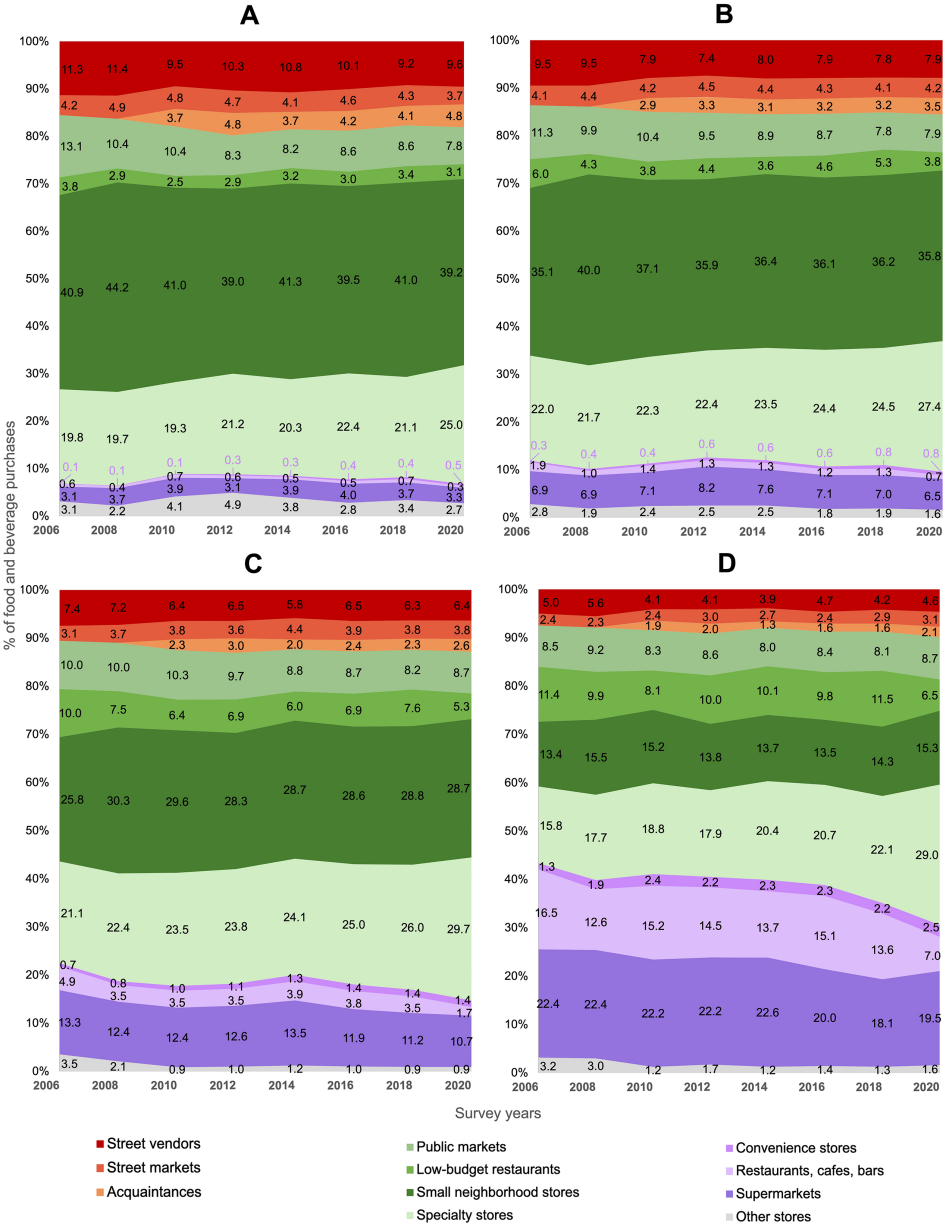
Trends in food purchases (% expenses) by outlet type according to the educational level of the head of the household: ENIGH 2006–2020. **(A)** Without formal education, **(B)** primary school, **(C)** high school, and **(D)** higher education. *Informal* outlets include street markets, street vendors, and acquaintances (red and orange); *mixed* outlets include small neighborhood stores, public markets, low-budget restaurants, and specialty stores (green); *formal* outlets include supermarkets, restaurants, cafes, bars, and chain convenience stores (purple).

Figure 3 shows the trends in household food purchases by outlet type stratified by education level (percentage data with standard errors are available in Supplementary Table 2). At baseline, households with lower education levels purchased a higher proportion of their foods and beverages at mixed outlets, compared to those with higher education levels. However, purchases from mixed outlets among households without formal education decreased over time (−2.4 p.p., 95% CI −4.6, −0.3), but increased 10.4 p.p. (95% CI 7.8, 12.9) for households with higher education. Trends from specific stores of the mixed outlet show that, purchases from public markets decreased 5.3 p.p. (95% CI −6.9, −3.7) among households without formal education and 3.4 p.p. (95% CI −4,1, −2.8) in households with primary school, but remained stable for households with higher education. Purchases from small neighborhood stores remained stable across education levels. Purchases at specialty stores increased over time, but the magnitude of the increment was higher for more educated households (13.2 p.p., 95% CI 11.5, 14.9), increasing the most from 2018 to 2020 (6.9 p.p., 95% CI 5.8, 8.0) in these households. In the informal sector, purchases of households without formal education were twice the proportion of purchases of households with higher education. Purchases in street markets and street vendors from the informal sector decreased 2.2 p.p. (95% CI −4.1, −0.4) among less educated households and remained stable among more educated households. This is explained by the decrease of 1.8 p.p. (95% CI −3.4, −0.2) in purchases from street vendors among less educated households. Yet, food purchases from acquaintances also increased in less educated households from 2010 to 2020 (1.1 p.p., 95% CI 0.2, 2.0). Overall, purchases from the formal sector remained stable among households without formal education, but decreased 1.8 p.p. (95% CI −3.9, 0.1) among households with higher education. Purchases at chain convenience stores increased for all education levels; yet, they still represent a very small proportion of overall purchases. Trend results from 1994 to 2020 show similar patterns and are presented in Supplementary Figure 1; Supplementary Table 4.

**Figure 4:**
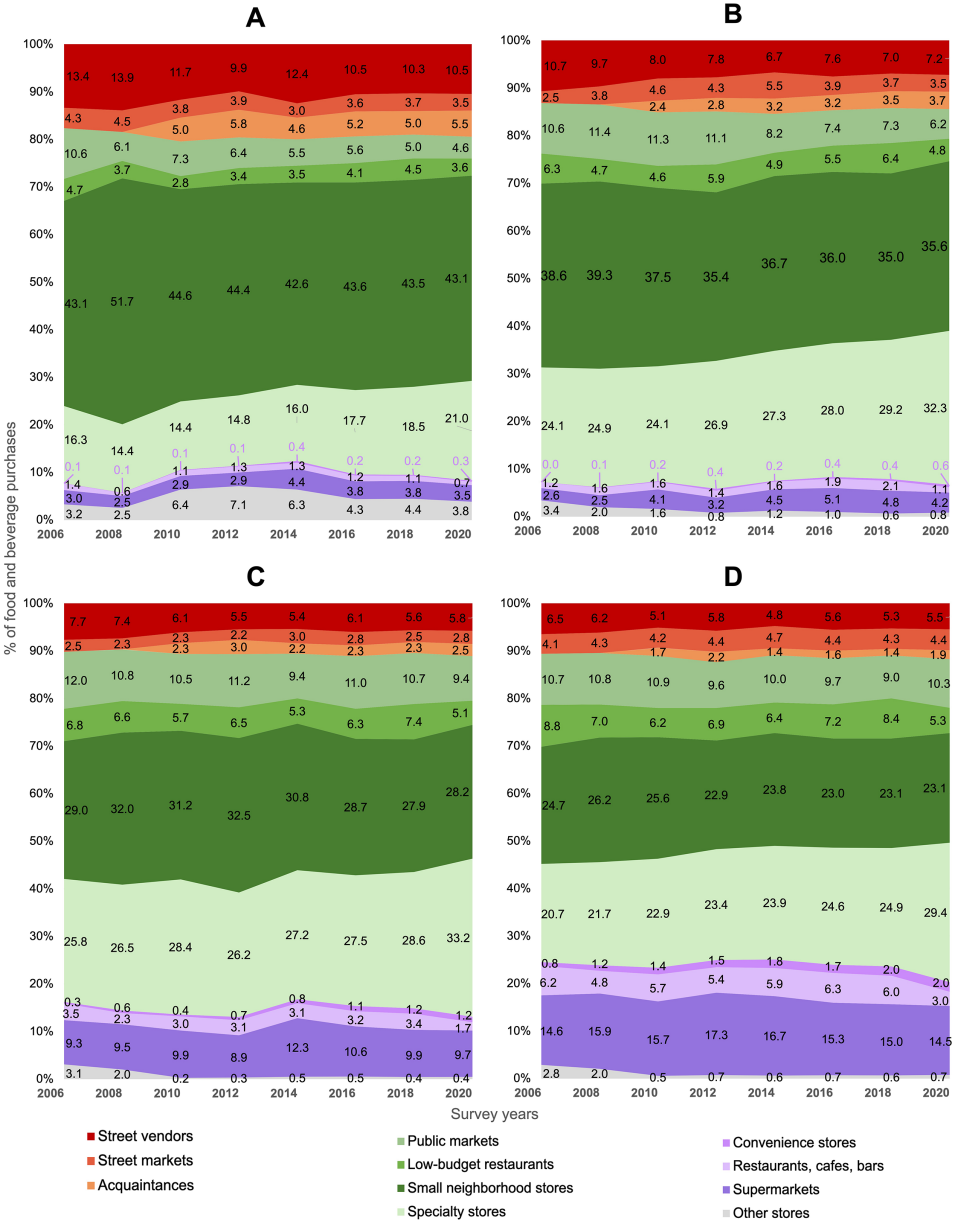
Trends in food purchases (% expenses) by outlet type according to the urbanicity: ENIGH 2006–2020. **(A)** Rural localities (<2,500 inhabitants), **(B)** small cities (2,500–14,999 inhabitants), **(C)** medium cities (15,000–99,999 inhabitants), and **(D)** metropolitan cities (>100,000 inhabitants). *Informal outlets* include street markets, street vendors and acquaintances (red and orange); *mixed outlets* include small neighborhood stores, public markets, low-budget restaurants, and specialty stores (green); *formal outlets* include supermarkets, restaurants, cafes, bars, and chain convenience stores (purple).

Figure 4 shows trends in household purchases in food and beverages by outlet type, stratified by urbanicity (percentage data with standard errors are available in Supplementary Table 3). In 2006, households in rural localities purchased most of their foods in mixed outlets, decreasing 2.4 p.p. (95% CI −3.8, −0.9) over time. In contrast, households living in metropolitan cities increased 3.1 p.p. (95% CI 2.1, 4.1) their purchases in mixed outlets from 2006 to 2020. Purchases from small neighborhood stores remained stable over time across localities, except in small cities, where purchases decreased 3.0 p.p. (95% CI −5.0, −0.9). Food purchases in public markets decreased 6.0 p.p. (95% CI −7.0, −5.1) among households in rural localities, but remained stable in households living in metropolitan cities. Purchases at specialty stores increased from 2006 to 2020, however, the largest increment was observed in metropolitan cities (8.7 p.p., 95% CI 7.8, 9.6) in contrast to rural localities (4.7 p.p., 95% CI 3.5, 5.9). Over time, rural localities relied more on the informal sector than metropolitan cities. Food purchases in street markets and street vendors from the informal sector decreased 0.8 p.p. (95% CI −1.4, −0.1) and 2.9 p.p. (95% CI −4.0, −1.8) respectively from 2006 to 2020 in rural localities. Trends in the formal sector (without considering restaurants, bars and cafes) showed a small increase in both, rural localities and metropolitan cities. Purchases in supermarkets remained stable across localities, except in small cities, where purchases increased in 1.7 p.p. (95% CI 1.0, 2.4). Regardless of urbanicity, purchases in convenience stores increased. Trend results from 1994 to 2020 show similar patterns and are presented in Supplementary Figure 2; Supplementary Table 5.

## 4. Discussion

Our study provides a deep understanding of the role that the mixed, informal, and formal sectors have played over time in food and beverage purchases for the Mexican population, for different socioeconomic sectors and locality sizes. We found that over time, the highest proportion of purchases were made at small neighborhood stores and specialty stores, followed by public markets. One of the most important changes in the mixed food sector was the increase in the contribution of specialty stores, which showed the largest increment in households with higher education and those living in metropolitan cities. Another important finding was the overall decrease in purchases from public markets, showing the largest decrease among households with lower educational levels and those living in rural localities. Street vendors accounted for most of the food purchases from the informal food sector. However, purchases at these outlets decreased over time, especially among households with lower educational levels and in rural localities. In contrast, purchases from street markets remained stable. Regarding the formal food sector, supermarkets represented the most important source of food purchases in this sector and remained stable over time. Trends by education showed that food purchases in supermarkets decreased among households with higher education and remained stable in households without formal education. Food purchases at restaurants, bars, and cafes were also stable over time, until 2020, when they showed an important decrease in 2020. This could be explained by COVID-19 pandemic. Starting in 2020, restaurants, bars, and cafes were either closed or had limited hours of operations by a national mandate. These restrictions had an important impact in social behavior and thus, in food purchases (22, 23). There was an overall increase in purchases from chain convenience stores, however, they still represent a minimum proportion of the total purchases across all education levels and localities.

Our study showed an overall increase in food purchases from specialty stores and chain convenience stores, and a slight decrease in food purchases from small neighborhood stores. However, we show that over time, purchases from small neighborhood and specialty stores are still higher, compared to purchases from supermarkets and chain convenience stores. Two recent studies (15, 16) that used Mexico’s economic census data (National Statistical Directory of Economic Units, DENUE) from 2010 to 2020 found a decrease of 12% in the number of small neighborhood stores at the municipality level (15, 16). While purchases from small neighborhood stores decreased over time, the decrease in the number of outlets seems higher than the overall decrease in purchases in this type of outlet. The same studies (15, 16) found an increase in the number of specialty stores of up to 22% over the same period. This trend is in line with the increasing trend of purchases from specialty stores in our sample. Interestingly, the study by Ramirez-Toscano et al. reports a 77.5% increase in the number of chain convenience stores, and 80% increase in the number of supermarkets at the municipality level from 2010 to 2016 (15). Our results show that despite the increase in the number of supermarkets, purchases do not follow the same trend. It is likely that, over the period studied, increases in the availability of supermarkets has not been translated to increases in food purchases.

Food purchases in supermarkets and chain convenience stores increased the most in rural localities and small cities. However, these households have relied over time on the mixed and informal sector for most of their food purchases. In contrast, supermarkets have represented one of the food outlets in which over time, households with higher education and living in metropolitan cities have made most of their food purchases. These households also showed the largest increase in purchases from specialty stores and small neighborhood stores. In line with our results, Ramírez-Toscano et al. (16) found that in Mexico, non-urban areas had the largest increase in chain convenience stores, supermarkets, and specialty stores. This study also found that the number of small neighborhood stores slightly decreased in both, urban and non-urban areas (16). Similarly, a previous study that used data from Mexico’s Nielsen Consumer Panel from 2012 to 2015 documented that low socioeconomic households obtained most of their foods from traditional retailers (outlets from the mixed sector in our study), while high socioeconomic households shopped more at supermarkets (24). However, traditional retailers were not differentiated in that study, and when analyzed separately, we found that specialty stores are also an important source of food for households with higher education levels and have increased significantly over time.

Our results show that, over the years, the informal food sector has been an important source of food purchases for Mexican households, especially for those residing in smaller localities and those with lower education levels. Yet, households living in cities and with higher levels of education, also rely on the informal food sector for their food and beverage purchases. In fact, purchases from street markets in these groups increased over time. However, trends show an overall decline in purchases from street vendors, and a decline in purchases from street markets among households living in rural localities and those without formal education. This could be explained by the fact that local governments have favored the promotion and expansion of formal outlets over informal ones (25), and by the expansion of supermarkets and chain convenience stores from metropolitan cities to small cities and rural areas (11–13, 26). In order to generate healthy and equitable food environments in Mexico, local authorities should consider informal retailers as part of their development initiatives (26–28). Over the years, the informal food sector has provided autonomy and a source of income to marginalized populations, while contributing to a fair distribution of local resources in LMIC (26, 27, 29, 30). Women in poor communities have particularly taken advantage of the informal food sector to contribute to their families’ food security (26, 27). For consumers, the informal food sector offers culturally appropriate food at convenient locations and affordable prices (27). However, informal outlets also have problems in terms of food safety and access to potable water, particularly outlets where street food is prepared (26). Additionally, since the informal sector is not regulated, it does not comply with tax laws and does not pay social security to its employees (26, 27). Thus, local governments need to recognize the importance of the informal food sector as well as the challenges that come with it. Given the lack of regulation, informal outlets are a more complex area to intervene in contrast to the formal food sector (3, 28).

For the last two decades, the mixed sector has represented the largest proportion of food and beverage expenditure in Mexico. Over time, there was an increase in food and beverage purchases in specialty stores, especially in larger cities, and an important decrease in purchases from public markets. Overall, small neighborhood stores remained stable and an important food source over time. The increase in purchases from specialty stores can be explained by the increase in the number of these food outlets (16). Also, the three most purchased food items in Mexico City are tortillas, fresh chicken, and vegetables, which most households tend to purchase in specialty stores (31). It is important to note that the highest increment in food and beverage purchases from specialty stores happened from 2018 to 2020, which could be related to the COVID-19 pandemic. A possible explanation for this increase could be that specialty stores are usually located at street level, and are smaller than other types of stores. Thus, people might have perceived less danger purchasing foods in specialty stores, than small-neighborhood stores or supermarkets. The decrease in purchases from public markets could be partially explained by the fact that these outlets depend on government funding; thus, as funding has decreased, public markets may have lacked maintenance, hygiene, and good infrastructure for food preservation (32). It has been shown that food quality, freshness, price and diversity of products are important reasons for people to keep purchasing their food at public markets (32). However, because public markets are outlets with high availability of fresh and natural foods, it is of concern that households with lower socioeconomic levels are purchasing less in these outlets. Regardless of socioeconomic level, households purchase an important proportion of their foods in small neighborhood stores. Yet, over time, these food outlets have been the most important food source for households in rural localities and with lower education levels. Households in lower socioeconomic levels find small neighborhood stores convenient, since they tend to be at a walking distance from their homes and people can purchase smaller amounts of foods (24, 29, 31, 33). Moreover, mixed outlets in Latin America, especially small neighborhood stores, had to evolve in order to compete with the modern sector. Around the 1990s, these outlets shifted to self-service and increased the diversity of their products, since they started using the food industry as suppliers of their products (17, 34, 35). Unfortunately, this supply includes a high proportion of sugary drinks, snacks, and sweets. Historically, when it comes to public health interventions, outlets from the mixed sector have been overlooked. The food policy agenda should start considering strategies to regulate small neighborhood stores’ food supply and distribution, while maintaining purchases from specialty stores, and increasing local governments’ funds directed toward the improvement of the environment within public markets.

The number of formal outlets in Mexico has increased over time (15, 16). We found that food purchases in supermarkets had a slight increase over time, while in chain convenience stores purchases doubled. Given that a higher density of chain convenience stores have been associated with poor nutrition and health outcomes such as diabetes (15), the rapid increase in food purchases in chain convenience stores could represent a threat to the population’s nutrition state. However, food purchases in chain convenience stores still represent a very low proportion of total food and beverage expenditure. A recent review documented that in some Latin American countries, including Mexico, the growth in the number of supermarkets happened from the late 1990s to mid 2000s, increasing from a 5–10% to a 30–50% (53% in Mexico) (3). Additionally, previous studies have argued that the expansion of chain convenience stores and supermarkets in LMIC might threaten the informal and mixed sectors (11–13, 26). While households with higher education and residing in metropolitan cities have bought a higher proportion of their foods and beverages in supermarkets, these same households increased their purchases in specialty stores and small neighborhood stores over time. On the other hand, households in smaller localities still purchase most of their foods in the mixed sector, although purchases in the formal sector in less urbanized localities increased over time. Supermarkets and chain convenience stores have been the target of many policies aimed at increasing the healthfulness of the retail food environment (36). However, only focusing policies on these types of outlets in Mexico would mostly benefit the segments of the population that purchase most of their foods there, which are households with a higher education level and those who reside in metropolitan cities.

Food policies targeting the retail food environment face important challenges. Most of the interventions involving the food environment in Mexico have targeted consumers’ food choices (3, 24, 37, 38). Yet, very few interventions have focused on the retailers themselves or have considered retailers as policy actors for the modification of the retail food environment (6, 38). Currently, there are no policies in place targeting the retail food environment in Mexico. In fact, according to The INFORMAS Healthy Food Environment Policy index in Mexico, the level of implementation of interventions in food retailers is very low, compared to international practices (6). As our results show, to reach better health outcomes at the population level, interventions within specific outlet types are essential to the transformation of the retail food environment (3, 6, 26, 36, 39). Specifically, given the importance of the different outlet-types to total food and beverage purchases for different population sectors, our study highlights the need to prioritize interventions in small neighborhood stores, specialty stores, and public markets.

A major strength of our study was the use of a nationally representative survey to describe trends in purchases in the mixed, informal, and formal food sector in Mexico. However, some limitations should be considered. While ENIGH has a temporary coverage from 1984 to 2020, we excluded from the analysis surveys prior to 1994 since ENIGH’s oldest outlet classification did not allow us to distinguish formal sector food purchases from purchases made in the informal sector. Moreover, the name of the establishments where purchases were made are not provided by ENIGH. Thus, our classification of formal, mixed, and informal outlets is limited to the type of food outlet reported by ENIGH. As a result of this, we are not able to determine whether a specific mixed outlet tends more toward a formal or an informal establishment. Food and beverage purchases from certain outlets could be underreported, especially if purchases were not planned. However, there is no reason to believe that underreporting is differential over time. While it could be possible that household purchases to differ by education level within each urbanicity category, we did not consider this in our study and would be important to explore in future research. One limitation of the ENIGH food outlet classifications is that some stores, such as specialty stores and street vendors, could sell a wide variety of foods. Thus, the impact of the changes in the proportion of purchases in these types of store could be hard to interpret without knowing what they sell. This study is not capturing changes in the number of stores over time. Therefore, our results are a combination of food store availability and where households choose to shop. Additionally, since the COVID-19 pandemic shifted both, purchase behaviors and changed the food environment, especially with closures of businesses, the interpretation of the 2020 data should consider the impact of the COVID-19 pandemic. Finally, this study did not look at the food groups that households are purchasing at the different food outlets. Future studies are needed to understand the quality of food purchases by store type.

## 5. Conclusion

In conclusion, we found that over time, food purchases increased the most in specialty stores and chain convenience stores but decreased in public markets and street vendors. This decrease was more important among households with lower education levels and those living in rural localities. Even though we observed an increase in food purchases from the formal sector, the predominant source for food in Mexico continues to be the mixed sector. Future policies targeting the retail food environment should consider that all households, except the ones with formal education and residing in metropolitan cities, make most of their food purchases in small neighborhood stores, which is concerning, given that these outlets are mostly supplied by the soft drink and processed food industries. A worrisome finding is the decrease in purchases in public markets, particularly for lower socioeconomic levels and smaller localities, which could imply a reduction in the consumption of fresh produce that are the staple of these outlets.

## Data availability statement

The original contributions presented in the study are included in the article/Supplementary material, further inquiries can be directed to the corresponding author.

## Author contributions

AD-B conducted the statistical analysis. AD-B and DS designed the study, interpreted the results, and led the writing. IF, NL-O, CP-F, YR-T, and TB-G contributed to the interpretation of results and critical review of the manuscript. All authors read, edited, and approved the final version of the manuscript.

## Funding

This research was supported by the Salud Urbana en América Latina (SALURBAL)/Urban Health in Latin America project funded by the Wellcome Trust (205177/Z/16/Z).

## Conflict of interest

The authors declare that the research was conducted in the absence of any commercial or financial relationships that could be construed as a potential conflict of interest.

## Publisher’s note

All claims expressed in this article are solely those of the authors and do not necessarily represent those of their affiliated organizations, or those of the publisher, the editors and the reviewers. Any product that may be evaluated in this article, or claim that may be made by its manufacturer, is not guaranteed or endorsed by the publisher.
