# Supplementary material for: Trends in food and beverage purchases in informal, mixed, and formal food outlets in Mexico: ENIGH 1994–2020
Source: Front Public Health. 2023 May 24;11:1151916. doi: 10.3389/fpubh.2023.1151916 (PMC10244666; doi:10.3389/fpubh.2023.1151916)
Supplement: Supplementary file 1 [file Data_Sheet_1.docx]

Supplementary Material

# Supplementary Tables

## Supplementary Table 1. Outlet categorization in ENIGH 1984 – 2020

## Supplementary Table 2. Food and beverage purchases (% expenditure) by outlet type stratified by education level of the head of the household, ENIGH 2006 – 2020

## Supplementary Table 3. Food and beverage purchases (% expenditure) by store type stratified by urbanicity, ENIGH 2006 – 2020.

## Supplementary Table 4. Food and beverage purchases (% expenditure) by store type stratified by education level of the head of the household, ENIGH 1994 – 2020.

## Supplementary Table 5. Food and beverage purchases (% expenditure) by store type stratified by urbanicity, ENIGH 1994 – 2020.

# Supplementary Figures

## Supplementary Figure 1. Trends in food purchases (% expenses) by store stratified by education level of the head of the household, ENIGH 1994 - 2020.

## Supplementary Figure 2. Trends in food purchases (% expenses) by store type stratified by urbanicity, ENIGH 1994 - 2020.

# Supplementary Table 1. Outlet categorization in ENIGH 1984 - 2020

| **Year** | **1984** | **1989** | **1992** | **1994 - 2000** | **2002-2005** | **2006** | **2008** | **2010 - 2020** |
| --- | --- | --- | --- | --- | --- | --- | --- | --- |
| **1** | Public market or street market | Public market or street market | Public market or street market | Public market | Public market | Public market | Public market | Public market |
| **2** | Self-service stores | Supermarkets or Department stores | Abarrotes and small stores | Street markets and street vendors | Street markets and street vendors | Street markets (tianguis) | Street markets (tianguis) | Street markets (tianguis) |
| **3** | Specialty stores | Specialty stores | Specialty stores | Specialty stores | Specialty stores | Street vendors | Street vendors | Street vendors |
| **4** | CONASUPO | CONASUPO Y DICONSA | Self-service stores or Supermarkets | Supermarkets and department stores | Supermarkets and department stores | Small neighborhood store (abarrotes) | Small neighborhood store (abarrotes) | Small neighborhood store (abarrotes) |
| **5** | Stores for government employees | LICONSA | Department stores | Purchase outside the country | Purchase outside the country | Specialty stores | Specialty stores | Specialty stores |
| **6** | Stores for private initiative | I.M.S.S. | Imported articles stores | Restaurants | Restaurants | Supermarkets | Supermarkets | Supermarkets |
| **7** | Purchase outside the country | I.S.S.S.T .E. | Street vendors | Low budget restaurants | Low budget restaurants | Department stores | Department stores | Department stores |
| **8** | Street vendors | FONACOT | Social Service | Cafeterias | Cafeterias | Purchase outside the country | Purchase outside the country | Purchase outside the country |
| **9** |  | Social Service | Financial Institutions | Bar | Bar | Membership stores | Membership stores | Membership stores |
| **10** |  | Navy or Army | Acquaintances | Other | Other | Convenience stores | Convenience stores | Convenience stores |
| **11** |  | Universities | Medical services I.M.S.S | At work |  | Restaurants | Restaurants | Restaurants |
| **12** |  | D.D.F. Stores | Medical services I.S.S.S.T.E | At school |  | Low budget restaurants | Low budget restaurants | Low budget restaurants |
| **13** |  | Stores for private initiative employees | Medical services in state institutions |  |  | Cafeterias | Cafeterias | Low budget restaurants |
| **14** |  | Cooperativas | Private medical services |  |  | Bar | Bar | Bar |
| **15** |  | Purchase outside the country | Medical dispensaries |  |  | Other | Other | Diconsa |
| **16** |  | Others | Healer, herbalist, empirical services |  |  |  |  | Lechería Liconsa |
| **17** |  |  | Other |  |  |  |  | Acquaintances |
| **18** |  |  |  |  |  |  |  | Internet |
|  |  |  |  |  |  |  |  |  |

**
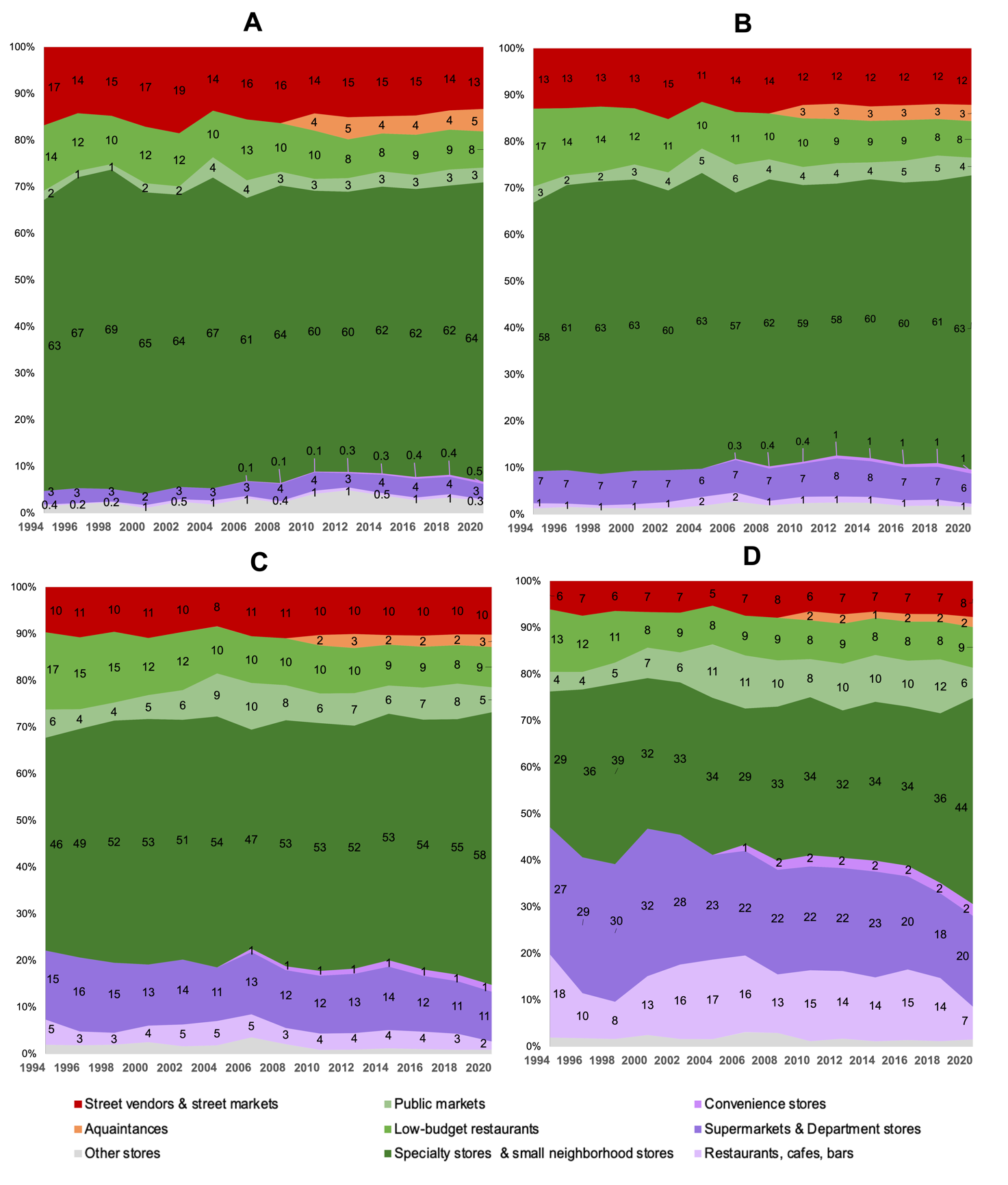
Supplementary Figure 1. Trends in food purchases (% expenses) by store stratified by education level of the head of the household, ENIGH 1994 - 2020.** (A) Without formal education, (B) Primary school, (C) High school, (D) Higher Education. *Informal outlets* include street vendors & street markets and acquaintances (red and orange); *mixed outlets* include public markets, low-budget restaurants, and specialty stores & small neighborhood stores, (green); *formal outlets* include supermarkets, restaurants, cafes, bars, and chain convenience stores (purple).

**
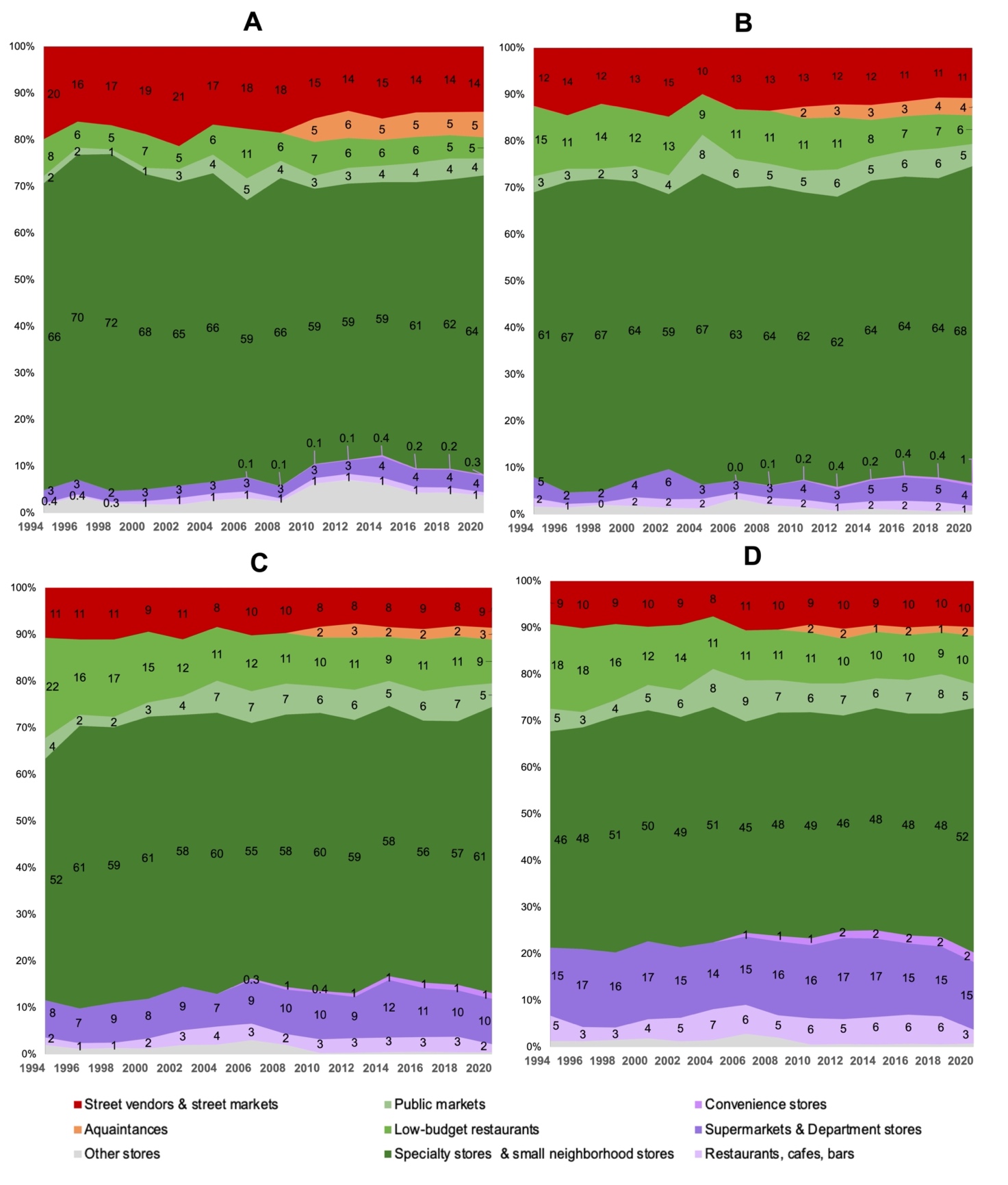
Supplementary Figure 2. Trends in food purchases (% expenses) by store type stratified by urbanicity, ENIGH 1994 - 2020.** (A) Rural localities (< 2,500 inhabitants), (B) Small cities (2,500 – 14,999 inhabitants), (C) Medium cities (15,000 – 99,999 inhabitants), (D) Metropolitan cities (> 100,000 inhabitants)*. Informal outlets* include street vendors & street markets and acquaintances (red and orange); *mixed outlets* include public markets, low-budget restaurants, and specialty stores & small neighborhood stores, (green); *formal outlets* include supermarkets, restaurants, cafes, bars, and chain convenience stores (purple).

**Supplementary Table 2.** Food and beverage purchases (% expenditure) by outlet type stratified by education level of the head of the household, ENIGH 2006 – 2020

| Education Level | Food Outlets | 2006 | | 2008 | | 2010 | | 2012 | | 2014 | | 2016 | | 2018 | | 2020 | |
| --- | --- | --- | --- | --- | --- | --- | --- | --- | --- | --- | --- | --- | --- | --- | --- | --- | --- |
|  |  | % | SE | % | SE | % | SE | % | SE | % | SE | % | SE | % | SE | % | SE |
| Without formal education | Street markets | 4.18 | 0.47 | 4.87 | 0.52 | 4.76 | 0.64 | 4.72 | 0.68 | 4.08 | 0.46 | 4.59 | 0.30 | 4.34 | 0.31 | 3.71 | 0.27 |
|  | Street vendors | 11.34 | 0.68 | 11.45 | 0.67 | 9.46 | 0.62 | 10.35 | 0.87 | 10.77 | 0.65 | 10.09 | 0.34 | 9.24 | 0.32 | 9.57 | 0.32 |
|  | Acquaintances | - | - | - | - | 3.71 | 0.31 | 4.75 | 0.58 | 3.68 | 0.36 | 4.16 | 0.23 | 4.13 | 0.24 | 4.80 | 0.24 |
|  | Low-budget restaurants | 3.75 | 0.35 | 2.94 | 0.29 | 2.51 | 0.37 | 2.88 | 0.41 | 3.25 | 0.39 | 3.02 | 0.20 | 3.42 | 0.22 | 3.14 | 0.21 |
|  | Public markets | 13.10 | 1.36 | 10.45 | 0.67 | 10.37 | 0.66 | 8.32 | 0.99 | 8.16 | 0.75 | 8.61 | 0.46 | 8.58 | 0.47 | 7.79 | 0.40 |
|  | Specialty stores | 19.84 | 0.90 | 19.72 | 0.67 | 19.35 | 0.79 | 21.17 | 1.18 | 20.31 | 0.90 | 22.36 | 0.57 | 21.11 | 0.53 | 25.04 | 0.49 |
|  | Small neighborhood stores | 40.90 | 1.18 | 44.17 | 0.96 | 40.96 | 1.25 | 38.98 | 1.40 | 41.26 | 1.09 | 39.48 | 0.63 | 40.99 | 0.70 | 39.20 | 0.63 |
|  | Convenience stores | 0.10 | 0.05 | 0.13 | 0.05 | 0.14 | 0.04 | 0.28 | 0.11 | 0.26 | 0.07 | 0.35 | 0.07 | 0.38 | 0.07 | 0.50 | 0.07 |
|  | Supermarkets | 3.10 | 0.35 | 3.70 | 0.38 | 3.93 | 0.39 | 3.06 | 0.51 | 3.93 | 0.45 | 4.03 | 0.25 | 3.74 | 0.24 | 3.27 | 0.20 |
|  | Restaurants, cafes, bars | 0.57 | 0.10 | 0.41 | 0.09 | 0.67 | 0.12 | 0.59 | 0.20 | 0.49 | 0.10 | 0.55 | 0.07 | 0.71 | 0.11 | 0.29 | 0.06 |
|  | Other stores | 3.11 | 0.28 | 2.17 | 0.28 | 4.13 | 0.75 | 4.91 | 1.39 | 3.83 | 0.63 | 2.78 | 0.38 | 3.35 | 0.35 | 2.69 | 0.24 |
| Primary School | Street markets | 4.12 | 0.28 | 4.40 | 0.24 | 4.24 | 0.28 | 4.49 | 0.37 | 4.44 | 0.31 | 4.33 | 0.15 | 4.11 | 0.14 | 4.17 | 0.17 |
|  | Street vendors | 9.52 | 0.33 | 9.55 | 0.34 | 7.92 | 0.28 | 7.37 | 0.32 | 8.01 | 0.31 | 7.87 | 0.16 | 7.84 | 0.17 | 7.92 | 0.15 |
|  | Acquaintances | - | - | - | - | 2.86 | 0.16 | 3.30 | 0.24 | 3.09 | 0.17 | 3.19 | 0.10 | 3.20 | 0.10 | 3.49 | 0.09 |
|  | Low-budget restaurants | 6.00 | 0.23 | 4.26 | 0.15 | 3.84 | 0.19 | 4.37 | 0.31 | 3.61 | 0.17 | 4.61 | 0.12 | 5.35 | 0.14 | 3.79 | 0.11 |
|  | Public markets | 11.30 | 0.93 | 9.86 | 0.38 | 10.44 | 0.43 | 9.48 | 0.70 | 8.93 | 0.47 | 8.75 | 0.27 | 7.85 | 0.24 | 7.86 | 0.22 |
|  | Specialty stores | 22.01 | 0.41 | 21.66 | 0.37 | 22.34 | 0.41 | 22.41 | 0.67 | 23.48 | 0.48 | 24.41 | 0.29 | 24.46 | 0.29 | 27.37 | 0.26 |
|  | Small neighborhood stores | 35.13 | 0.68 | 39.99 | 0.50 | 37.08 | 0.59 | 35.94 | 0.91 | 36.37 | 0.62 | 36.09 | 0.32 | 36.17 | 0.31 | 35.80 | 0.29 |
|  | Convenience stores | 0.25 | 0.03 | 0.41 | 0.05 | 0.38 | 0.05 | 0.63 | 0.09 | 0.65 | 0.07 | 0.59 | 0.03 | 0.81 | 0.04 | 0.83 | 0.04 |
|  | Supermarkets | 6.95 | 0.28 | 6.94 | 0.28 | 7.08 | 0.31 | 8.16 | 0.55 | 7.64 | 0.38 | 7.11 | 0.19 | 7.02 | 0.18 | 6.46 | 0.16 |
|  | Restaurants, cafes, bars | 1.93 | 0.12 | 1.01 | 0.07 | 1.38 | 0.09 | 1.31 | 0.15 | 1.25 | 0.11 | 1.24 | 0.06 | 1.27 | 0.06 | 0.68 | 0.04 |
|  | Other stores | 2.79 | 0.15 | 1.91 | 0.16 | 2.44 | 0.30 | 2.54 | 0.48 | 2.54 | 0.27 | 1.81 | 0.11 | 1.92 | 0.12 | 1.62 | 0.10 |
| High School | Street markets | 3.09 | 0.20 | 3.73 | 0.16 | 3.82 | 0.22 | 3.58 | 0.29 | 4.44 | 0.24 | 3.91 | 0.13 | 3.78 | 0.11 | 3.84 | 0.12 |
|  | Street vendors | 7.45 | 0.28 | 7.24 | 0.21 | 6.42 | 0.20 | 6.46 | 0.35 | 5.85 | 0.22 | 6.47 | 0.13 | 6.32 | 0.13 | 6.36 | 0.11 |
|  | Acquaintances | - | - | - | - | 2.26 | 0.11 | 3.01 | 0.31 | 2.05 | 0.11 | 2.40 | 0.07 | 2.33 | 0.07 | 2.62 | 0.06 |
|  | Low-budget restaurants | 10.01 | 0.40 | 7.52 | 0.23 | 6.36 | 0.22 | 6.95 | 0.42 | 6.04 | 0.24 | 6.85 | 0.15 | 7.59 | 0.15 | 5.33 | 0.12 |
|  | Public markets | 10.00 | 0.49 | 10.04 | 0.32 | 10.29 | 0.32 | 9.67 | 0.64 | 8.75 | 0.38 | 8.74 | 0.25 | 8.25 | 0.23 | 8.67 | 0.22 |
|  | Specialty stores | 21.13 | 0.49 | 22.38 | 0.34 | 23.51 | 0.39 | 23.81 | 0.61 | 24.14 | 0.42 | 24.99 | 0.24 | 25.97 | 0.28 | 29.70 | 0.23 |
|  | Small neighborhood stores | 25.84 | 0.58 | 30.34 | 0.41 | 29.59 | 0.45 | 28.29 | 0.66 | 28.70 | 0.47 | 28.59 | 0.25 | 28.82 | 0.25 | 28.73 | 0.22 |
|  | Convenience stores | 0.68 | 0.08 | 0.80 | 0.08 | 1.01 | 0.10 | 1.07 | 0.11 | 1.34 | 0.10 | 1.35 | 0.05 | 1.39 | 0.05 | 1.42 | 0.04 |
|  | Supermarkets | 13.31 | 0.51 | 12.38 | 0.34 | 12.36 | 0.37 | 12.63 | 0.69 | 13.52 | 0.45 | 11.91 | 0.22 | 11.21 | 0.22 | 10.66 | 0.18 |
|  | Restaurants, cafes, bars | 4.95 | 0.28 | 3.48 | 0.20 | 3.53 | 0.17 | 3.53 | 0.30 | 3.93 | 0.20 | 3.77 | 0.12 | 3.45 | 0.10 | 1.75 | 0.07 |
|  | Other stores | 3.54 | 0.27 | 2.08 | 0.13 | 0.86 | 0.09 | 1.00 | 0.13 | 1.23 | 0.12 | 1.02 | 0.05 | 0.90 | 0.05 | 0.92 | 0.04 |
| Higher education | Street markets | 2.43 | 0.25 | 2.31 | 0.17 | 2.37 | 0.20 | 2.97 | 0.52 | 2.66 | 0.24 | 2.43 | 0.18 | 2.89 | 0.18 | 3.09 | 0.18 |
|  | Street vendors | 5.02 | 0.33 | 5.57 | 0.29 | 4.12 | 0.21 | 4.15 | 0.38 | 3.91 | 0.24 | 4.66 | 0.18 | 4.20 | 0.18 | 4.64 | 0.16 |
|  | Acquaintances | - | - | - | - | 1.94 | 0.19 | 2.05 | 0.41 | 1.34 | 0.14 | 1.62 | 0.09 | 1.62 | 0.09 | 2.14 | 0.10 |
|  | Low-budget restaurants | 11.38 | 0.55 | 9.90 | 0.42 | 8.14 | 0.43 | 10.02 | 0.87 | 10.05 | 0.64 | 9.84 | 0.34 | 11.53 | 0.34 | 6.49 | 0.24 |
|  | Public markets | 8.54 | 0.57 | 9.16 | 0.49 | 8.35 | 0.43 | 8.59 | 0.98 | 7.99 | 0.56 | 8.40 | 0.35 | 8.14 | 0.34 | 8.75 | 0.34 |
|  | Specialty stores | 15.85 | 0.56 | 17.66 | 0.53 | 18.78 | 0.57 | 17.91 | 0.99 | 20.41 | 0.64 | 20.72 | 0.39 | 22.12 | 0.40 | 29.01 | 0.42 |
|  | Small neighborhood stores | 13.38 | 0.49 | 15.51 | 0.53 | 15.19 | 0.56 | 13.75 | 0.87 | 13.71 | 0.54 | 13.49 | 0.30 | 14.34 | 0.30 | 15.27 | 0.27 |
|  | Convenience stores | 1.34 | 0.18 | 1.91 | 0.28 | 2.41 | 0.35 | 2.18 | 0.41 | 2.34 | 0.21 | 2.26 | 0.13 | 2.22 | 0.10 | 2.49 | 0.11 |
|  | Supermarkets | 22.40 | 0.86 | 22.42 | 0.76 | 22.24 | 0.85 | 22.15 | 1.47 | 22.61 | 0.90 | 20.01 | 0.51 | 18.11 | 0.42 | 19.47 | 0.44 |
|  | Restaurants, cafes, bars | 16.50 | 0.74 | 12.56 | 0.61 | 15.24 | 0.75 | 14.50 | 1.27 | 13.73 | 0.71 | 15.14 | 0.45 | 13.56 | 0.45 | 7.05 | 0.31 |
|  | Other stores | 3.16 | 0.26 | 2.99 | 0.30 | 1.22 | 0.15 | 1.72 | 0.33 | 1.25 | 0.17 | 1.43 | 0.12 | 1.26 | 0.10 | 1.60 | 0.13 |

**Supplementary Table 3.** Food and beverage purchases (% expenditure) by store type stratified by urbanicity, ENIGH 2006 – 2020.

| Urbanicity | Food Outlets | 2006 | | 2008 | | 2010 | | 2012 | | 2014 | | 2016 | | 2018 | | 2020 | |
| --- | --- | --- | --- | --- | --- | --- | --- | --- | --- | --- | --- | --- | --- | --- | --- | --- | --- |
|  |  | % | SE | % | SE | % | SE | % | SE | % | SE | % | SE | % | SE | % | SE |
| Rural localities | Street markets | 4.27 | 0.64 | 4.54 | 0.59 | 3.76 | 0.65 | 3.88 | 0.62 | 3.01 | 0.41 | 3.61 | 0.23 | 3.74 | 0.23 | 3.50 | 0.25 |
|  | Street vendors | 13.38 | 0.88 | 13.92 | 0.81 | 11.67 | 0.72 | 9.90 | 0.67 | 12.43 | 0.69 | 10.53 | 0.31 | 10.32 | 0.28 | 10.47 | 0.25 |
|  | Acquaintances | - | - | - | - | 5.01 | 0.41 | 5.83 | 0.56 | 4.58 | 0.30 | 5.23 | 0.19 | 4.95 | 0.17 | 5.47 | 0.15 |
|  | Low-budget restaurants | 4.66 | 0.34 | 3.67 | 0.25 | 2.76 | 0.24 | 3.37 | 0.30 | 3.51 | 0.25 | 4.09 | 0.15 | 4.49 | 0.13 | 3.63 | 0.11 |
|  | Public markets | 10.63 | 2.67 | 6.06 | 0.56 | 7.28 | 0.66 | 6.42 | 0.94 | 5.54 | 0.54 | 5.61 | 0.31 | 5.00 | 0.25 | 4.58 | 0.24 |
|  | Specialty stores | 16.27 | 1.04 | 14.41 | 0.63 | 14.39 | 0.73 | 14.79 | 0.86 | 16.00 | 0.69 | 17.75 | 0.38 | 18.49 | 0.37 | 20.98 | 0.36 |
|  | Small neighborhood stores | 43.11 | 2.05 | 51.70 | 1.06 | 44.63 | 1.30 | 44.44 | 1.47 | 42.56 | 1.04 | 43.64 | 0.55 | 43.52 | 0.51 | 43.12 | 0.48 |
|  | Convenience stores | 0.06 | 0.01 | 0.06 | 0.01 | 0.14 | 0.08 | 0.14 | 0.03 | 0.37 | 0.16 | 0.22 | 0.02 | 0.23 | 0.02 | 0.26 | 0.02 |
|  | Supermarkets | 3.01 | 0.44 | 2.52 | 0.29 | 2.87 | 0.40 | 2.86 | 0.47 | 4.40 | 0.48 | 3.76 | 0.18 | 3.77 | 0.18 | 3.52 | 0.14 |
|  | Restaurants, cafes, bars | 1.35 | 0.17 | 0.62 | 0.07 | 1.13 | 0.16 | 1.29 | 0.21 | 1.26 | 0.19 | 1.21 | 0.09 | 1.06 | 0.06 | 0.67 | 0.04 |
|  | Other stores | 3.25 | 0.32 | 2.49 | 0.21 | 6.36 | 0.87 | 7.08 | 1.35 | 6.35 | 0.71 | 4.35 | 0.30 | 4.42 | 0.28 | 3.79 | 0.23 |
| Small cities | Street markets | 2.46 | 0.39 | 3.77 | 0.51 | 4.64 | 1.08 | 4.31 | 0.84 | 5.51 | 0.86 | 3.87 | 0.38 | 3.70 | 0.28 | 3.52 | 0.43 |
|  | Street vendors | 10.70 | 0.80 | 9.67 | 0.68 | 8.02 | 0.53 | 7.77 | 0.73 | 6.72 | 0.37 | 7.60 | 0.30 | 6.97 | 0.31 | 7.25 | 0.30 |
|  | Acquaintances | - | - | - | - | 2.44 | 0.20 | 2.82 | 0.42 | 3.20 | 0.32 | 3.19 | 0.19 | 3.54 | 0.25 | 3.66 | 0.19 |
|  | Low-budget restaurants | 6.31 | 0.77 | 4.72 | 0.39 | 4.61 | 0.58 | 5.87 | 0.55 | 4.91 | 0.38 | 5.51 | 0.27 | 6.37 | 0.26 | 4.76 | 0.22 |
|  | Public markets | 10.61 | 1.97 | 11.44 | 1.21 | 11.26 | 1.15 | 11.14 | 2.07 | 8.15 | 0.99 | 7.43 | 0.70 | 7.35 | 0.62 | 6.19 | 0.43 |
|  | Specialty stores | 24.09 | 1.42 | 24.92 | 0.96 | 24.15 | 1.05 | 26.85 | 1.33 | 27.29 | 1.01 | 28.05 | 0.63 | 29.23 | 0.74 | 32.27 | 0.57 |
|  | Small neighborhood stores | 38.60 | 1.68 | 39.34 | 1.13 | 37.47 | 1.50 | 35.41 | 1.72 | 36.70 | 1.07 | 36.02 | 0.71 | 34.97 | 0.73 | 35.64 | 0.69 |
|  | Convenience stores | 0.03 | 0.01 | 0.10 | 0.04 | 0.15 | 0.06 | 0.43 | 0.16 | 0.22 | 0.07 | 0.38 | 0.06 | 0.40 | 0.05 | 0.57 | 0.06 |
|  | Supermarkets | 2.57 | 0.43 | 2.49 | 0.33 | 4.12 | 1.06 | 3.19 | 0.69 | 4.49 | 0.62 | 5.06 | 0.44 | 4.78 | 0.41 | 4.24 | 0.34 |
|  | Restaurants, cafes, bars | 1.24 | 0.24 | 1.56 | 0.34 | 1.57 | 0.21 | 1.45 | 0.40 | 1.57 | 0.20 | 1.93 | 0.18 | 2.06 | 0.15 | 1.08 | 0.09 |
|  | Other stores | 3.39 | 0.45 | 1.97 | 0.33 | 1.57 | 0.52 | 0.77 | 0.22 | 1.24 | 0.35 | 0.96 | 0.13 | 0.63 | 0.07 | 0.81 | 0.12 |
| Medium cities | Street markets | 2.55 | 0.34 | 2.32 | 0.21 | 2.30 | 0.24 | 2.18 | 0.45 | 3.03 | 0.37 | 2.79 | 0.25 | 2.49 | 0.22 | 2.75 | 0.27 |
|  | Street vendors | 7.66 | 0.39 | 7.38 | 0.35 | 6.07 | 0.30 | 5.48 | 0.52 | 5.38 | 0.37 | 6.06 | 0.24 | 5.65 | 0.30 | 5.79 | 0.21 |
|  | Acquaintances | - | - | - | - | 2.29 | 0.20 | 3.02 | 0.47 | 2.18 | 0.24 | 2.28 | 0.14 | 2.29 | 0.14 | 2.54 | 0.12 |
|  | Low-budget restaurants | 6.79 | 0.41 | 6.63 | 0.33 | 5.67 | 0.37 | 6.46 | 0.75 | 5.34 | 0.43 | 6.32 | 0.29 | 7.45 | 0.31 | 5.07 | 0.22 |
|  | Public markets | 11.96 | 1.36 | 10.83 | 0.83 | 10.48 | 0.81 | 11.18 | 1.56 | 9.38 | 0.88 | 11.04 | 0.69 | 10.71 | 0.62 | 9.39 | 0.49 |
|  | Specialty stores | 25.85 | 0.75 | 26.45 | 0.62 | 28.41 | 0.82 | 26.15 | 1.40 | 27.15 | 1.03 | 27.48 | 0.58 | 28.64 | 0.65 | 33.21 | 0.52 |
|  | Small neighborhood stores | 29.00 | 0.86 | 32.00 | 0.73 | 31.23 | 0.90 | 32.49 | 1.88 | 30.78 | 1.27 | 28.72 | 0.61 | 27.91 | 0.54 | 28.16 | 0.51 |
|  | Convenience stores | 0.33 | 0.08 | 0.55 | 0.13 | 0.39 | 0.08 | 0.72 | 0.25 | 0.83 | 0.17 | 1.10 | 0.12 | 1.16 | 0.12 | 1.17 | 0.09 |
|  | Supermarkets | 9.32 | 0.77 | 9.50 | 0.68 | 9.94 | 0.64 | 8.88 | 1.20 | 12.33 | 1.14 | 10.55 | 0.53 | 9.91 | 0.54 | 9.74 | 0.45 |
|  | Restaurants, cafes, bars | 3.49 | 0.25 | 2.33 | 0.19 | 2.97 | 0.27 | 3.13 | 0.80 | 3.13 | 0.34 | 3.18 | 0.21 | 3.38 | 0.23 | 1.74 | 0.13 |
|  | Other stores | 3.05 | 0.32 | 2.00 | 0.19 | 0.25 | 0.04 | 0.30 | 0.12 | 0.46 | 0.11 | 0.50 | 0.08 | 0.42 | 0.05 | 0.42 | 0.05 |
| Metropolitan cities | Street markets | 4.12 | 0.18 | 4.28 | 0.15 | 4.23 | 0.15 | 4.44 | 0.36 | 4.68 | 0.27 | 4.39 | 0.13 | 4.27 | 0.13 | 4.39 | 0.14 |
|  | Street vendors | 6.49 | 0.18 | 6.17 | 0.13 | 5.13 | 0.14 | 5.78 | 0.33 | 4.77 | 0.15 | 5.57 | 0.13 | 5.30 | 0.14 | 5.48 | 0.12 |
|  | Acquaintances | - | - | - | - | 1.72 | 0.09 | 2.15 | 0.25 | 1.45 | 0.09 | 1.61 | 0.06 | 1.40 | 0.05 | 1.86 | 0.05 |
|  | Low-budget restaurants | 8.82 | 0.24 | 6.96 | 0.18 | 6.18 | 0.19 | 6.86 | 0.44 | 6.41 | 0.26 | 7.20 | 0.16 | 8.43 | 0.17 | 5.32 | 0.13 |
|  | Public markets | 10.74 | 0.37 | 10.83 | 0.28 | 10.92 | 0.30 | 9.63 | 0.66 | 9.99 | 0.49 | 9.69 | 0.27 | 9.04 | 0.27 | 10.25 | 0.27 |
|  | Specialty stores | 20.69 | 0.34 | 21.72 | 0.29 | 22.95 | 0.32 | 23.35 | 0.69 | 23.92 | 0.43 | 24.64 | 0.26 | 24.92 | 0.28 | 29.36 | 0.26 |
|  | Small neighborhood stores | 24.68 | 0.36 | 26.23 | 0.34 | 25.56 | 0.38 | 22.87 | 0.66 | 23.75 | 0.42 | 22.98 | 0.23 | 23.05 | 0.24 | 23.09 | 0.21 |
|  | Convenience stores | 0.77 | 0.06 | 1.16 | 0.09 | 1.43 | 0.14 | 1.50 | 0.14 | 1.79 | 0.10 | 1.72 | 0.06 | 1.98 | 0.06 | 2.02 | 0.06 |
|  | Supermarkets | 14.63 | 0.34 | 15.86 | 0.34 | 15.69 | 0.38 | 17.35 | 0.80 | 16.71 | 0.48 | 15.26 | 0.24 | 14.99 | 0.24 | 14.49 | 0.22 |
|  | Restaurants, cafes, bars | 6.22 | 0.24 | 4.77 | 0.20 | 5.67 | 0.23 | 5.37 | 0.39 | 5.93 | 0.29 | 6.25 | 0.18 | 5.99 | 0.19 | 2.99 | 0.11 |
|  | Other stores | 2.84 | 0.15 | 2.02 | 0.13 | 0.52 | 0.05 | 0.69 | 0.11 | 0.61 | 0.06 | 0.68 | 0.04 | 0.62 | 0.04 | 0.75 | 0.04 |

**Supplementary Table 4.** Food and beverage purchases (% expenditure) by store type stratified by education level of the head of the household, ENIGH 1994 – 2020

| Education Level | Food Outlets | 1994 | | 1996 | | 1998 | | 2000 | | 2002 | | 2004 | | 2006 | |
| --- | --- | --- | --- | --- | --- | --- | --- | --- | --- | --- | --- | --- | --- | --- | --- |
|  |  | % | SE | % | SE | % | SE | % | SE | % | SE | % | SE | % | SE |
| Without formal education | Street markets & street vendors | 16.79 | 1.11 | 14.19 | 0.78 | 14.75 | 1.17 | 17.10 | 1.35 | 18.74 | 1.53 | 13.67 | 1.18 | 15.52 | 0.76 |
|  | Acquaintances | - | - | - | - | - | - | - | - | - | - | - | - | - | - |
|  | Low-budget restaurants | 1.96 | 0.33 | 1.33 | 0.22 | 1.16 | 0.32 | 1.95 | 0.34 | 1.80 | 0.25 | 4.33 | 0.63 | 3.75 | 0.35 |
|  | Public markets | 14.04 | 1.44 | 12.40 | 1.02 | 10.37 | 1.16 | 12.12 | 1.57 | 11.55 | 1.18 | 9.99 | 1.13 | 13.10 | 1.36 |
|  | Specialty stores & small neighborhood stores | 62.50 | 1.64 | 66.75 | 1.25 | 68.56 | 1.46 | 64.74 | 1.67 | 63.50 | 1.41 | 66.71 | 1.66 | 60.74 | 1.38 |
|  | Convenience stores | - | - | - | - | - | - | - | - | - | - | - | - | 0.10 | 0.05 |
|  | Supermarkets & Department stores | 2.88 | 0.47 | 3.20 | 0.44 | 2.71 | 0.45 | 2.43 | 0.46 | 2.72 | 0.43 | 2.60 | 0.34 | 3.11 | 0.35 |
|  | Restaurants, cafes, bars | 0.40 | 0.10 | 0.22 | 0.07 | 0.15 | 0.07 | 0.63 | 0.25 | 0.49 | 0.13 | 0.89 | 0.16 | 0.57 | 0.10 |
|  | Other stores | 1.43 | 0.27 | 1.91 | 0.31 | 2.30 | 0.42 | 1.03 | 0.28 | 2.48 | 0.23 | 1.81 | 0.41 | 3.10 | 0.28 |
| Primary school | Street markets & street vendors | 12.92 | 0.54 | 12.82 | 0.49 | 12.50 | 0.54 | 12.86 | 0.61 | 15.16 | 0.93 | 11.46 | 0.74 | 13.64 | 0.34 |
|  | Acquaintances | - | - | - | - | - | - | - | - | - | - | - | - | - | - |
|  | Low-budget restaurants | 3.42 | 0.24 | 2.09 | 0.17 | 2.08 | 0.20 | 3.21 | 0.38 | 3.85 | 0.26 | 5.22 | 0.28 | 6.00 | 0.23 |
|  | Public markets | 16.75 | 1.19 | 14.41 | 0.82 | 13.97 | 0.91 | 12.05 | 0.96 | 11.46 | 0.69 | 10.05 | 0.57 | 11.30 | 0.93 |
|  | Specialty stores & small neighborhood stores | 57.66 | 1.08 | 61.19 | 0.91 | 62.80 | 0.96 | 62.55 | 0.92 | 60.06 | 0.93 | 63.50 | 0.86 | 57.14 | 0.81 |
|  | Convenience stores | - | - | - | - | - | - | - | - | - | - | - | - | 0.25 | 0.03 |
|  | Supermarkets & Department stores | 6.88 | 0.54 | 7.18 | 0.64 | 6.71 | 0.49 | 7.18 | 0.61 | 6.81 | 0.40 | 6.03 | 0.33 | 6.97 | 0.28 |
|  | Restaurants, cafes, bars | 1.11 | 0.15 | 0.71 | 0.10 | 0.58 | 0.08 | 0.88 | 0.12 | 1.34 | 0.16 | 1.93 | 0.16 | 1.93 | 0.12 |
|  | Other stores | 1.26 | 0.17 | 1.59 | 0.19 | 1.36 | 0.14 | 1.27 | 0.17 | 1.31 | 0.15 | 1.82 | 0.21 | 2.77 | 0.15 |
| High School | Street markets & street vendors | 9.67 | 0.57 | 10.77 | 0.55 | 9.55 | 0.43 | 10.89 | 0.58 | 9.62 | 0.43 | 8.43 | 0.31 | 10.53 | 0.36 |
|  | Acquaintances | - | - | - | - | - | - | - | - | - | - | - | - | - | - |
|  | Low-budget restaurants | 6.01 | 0.60 | 4.20 | 0.36 | 3.86 | 0.38 | 5.12 | 0.47 | 6.35 | 0.45 | 9.21 | 0.38 | 10.01 | 0.40 |
|  | Public markets | 16.57 | 1.38 | 15.40 | 1.01 | 15.19 | 0.84 | 12.24 | 0.98 | 12.46 | 0.81 | 10.08 | 0.39 | 10.00 | 0.49 |
|  | Specialty stores & small neighborhood stores | 45.61 | 1.42 | 49.01 | 1.29 | 51.90 | 0.96 | 52.63 | 1.31 | 51.40 | 1.05 | 53.77 | 0.63 | 46.97 | 0.78 |
|  | Convenience stores | - | - | - | - | - | - | - | - | - | - | - | - | 0.68 | 0.08 |
|  | Supermarkets & Department stores | 14.79 | 1.11 | 15.83 | 1.73 | 14.99 | 0.88 | 13.08 | 0.93 | 13.90 | 0.66 | 11.49 | 0.45 | 13.35 | 0.51 |
|  | Restaurants, cafes, bars | 5.39 | 0.68 | 2.98 | 0.33 | 2.55 | 0.25 | 3.56 | 0.39 | 4.67 | 0.37 | 5.20 | 0.26 | 4.95 | 0.28 |
|  | Other stores | 1.95 | 0.41 | 1.81 | 0.21 | 1.95 | 0.25 | 2.49 | 0.38 | 1.60 | 0.19 | 1.83 | 0.15 | 3.51 | 0.27 |
| Higher education | Street markets & street vendors | 6.08 | 0.85 | 7.45 | 0.81 | 6.41 | 0.57 | 6.65 | 0.89 | 6.80 | 0.67 | 5.30 | 0.35 | 7.45 | 0.41 |
|  | Acquaintances | - | - | - | - | - | - | - | - | - | - | - | - | - | - |
|  | Low-budget restaurants | 4.22 | 0.62 | 3.74 | 0.55 | 4.52 | 0.69 | 6.55 | 0.86 | 6.44 | 0.74 | 11.44 | 0.89 | 11.38 | 0.55 |
|  | Public markets | 13.42 | 2.24 | 12.09 | 1.37 | 11.11 | 1.06 | 7.66 | 1.20 | 8.54 | 0.90 | 8.35 | 0.52 | 8.54 | 0.57 |
|  | Specialty stores & small neighborhood stores | 29.14 | 2.74 | 36.09 | 1.92 | 38.79 | 1.83 | 32.36 | 2.96 | 32.71 | 2.37 | 33.75 | 0.97 | 29.23 | 0.81 |
|  | Convenience stores | - | - | - | - | - | - | - | - | - | - | - | - | 1.34 | 0.18 |
|  | Supermarkets & Department stores | 27.35 | 2.89 | 29.17 | 2.18 | 29.55 | 1.72 | 31.66 | 3.73 | 27.89 | 3.67 | 22.51 | 0.79 | 22.49 | 0.86 |
|  | Restaurants, cafes, bars | 17.87 | 3.51 | 9.66 | 1.02 | 7.99 | 0.99 | 12.65 | 1.40 | 16.02 | 1.99 | 17.07 | 0.73 | 16.50 | 0.74 |
|  | Other stores | 1.92 | 0.57 | 1.80 | 0.48 | 1.63 | 0.36 | 2.48 | 0.58 | 1.60 | 0.34 | 1.59 | 0.19 | 3.07 | 0.26 |

**Supplementary Table 4.** Continuation

| Education Level | Food Outlets | 2008 | | 2010 | | 2012 | | 2014 | | 2016 | | 2018 | | 2020 | |
| --- | --- | --- | --- | --- | --- | --- | --- | --- | --- | --- | --- | --- | --- | --- | --- |
|  |  | % | SE | % | SE | % | SE | % | SE | % | SE | % | SE | % | SE |
| Without formal education | Street markets & street vendors | 16.32 | 0.78 | 14.23 | 0.88 | 15.06 | 1.04 | 14.85 | 0.70 | 14.67 | 0.42 | 13.58 | 0.42 | 13.28 | 0.41 |
|  | Acquaintances | - | - | 3.71 | 0.31 | 4.75 | 0.58 | 3.68 | 0.36 | 4.16 | 0.23 | 4.13 | 0.24 | 4.80 | 0.24 |
|  | Low-budget restaurants | 2.94 | 0.29 | 2.51 | 0.37 | 2.88 | 0.41 | 3.25 | 0.39 | 3.02 | 0.20 | 3.42 | 0.22 | 3.14 | 0.21 |
|  | Public markets | 10.45 | 0.67 | 10.37 | 0.66 | 8.32 | 0.99 | 8.16 | 0.75 | 8.61 | 0.46 | 8.58 | 0.47 | 7.79 | 0.40 |
|  | Specialty stores & small neighborhood stores | 63.88 | 0.99 | 60.31 | 1.20 | 60.15 | 1.69 | 61.57 | 1.14 | 61.83 | 0.68 | 62.11 | 0.69 | 64.24 | 0.60 |
|  | Convenience stores | 0.13 | 0.05 | 0.14 | 0.04 | 0.28 | 0.11 | 0.26 | 0.07 | 0.35 | 0.07 | 0.38 | 0.07 | 0.50 | 0.07 |
|  | Supermarkets & Department stores | 3.71 | 0.38 | 3.95 | 0.39 | 3.06 | 0.51 | 3.97 | 0.45 | 4.05 | 0.25 | 3.78 | 0.24 | 3.33 | 0.20 |
|  | Restaurants, cafes, bars | 0.41 | 0.09 | 0.67 | 0.12 | 0.59 | 0.20 | 0.49 | 0.10 | 0.55 | 0.07 | 0.71 | 0.11 | 0.29 | 0.06 |
|  | Other stores | 2.17 | 0.28 | 4.11 | 0.75 | 4.90 | 1.39 | 3.79 | 0.63 | 2.75 | 0.38 | 3.31 | 0.35 | 2.63 | 0.24 |
| Primary school | Street markets & street vendors | 13.95 | 0.39 | 12.16 | 0.38 | 11.86 | 0.47 | 12.45 | 0.40 | 12.20 | 0.22 | 11.94 | 0.22 | 12.10 | 0.22 |
|  | Acquaintances | - | - | 2.86 | 0.16 | 3.30 | 0.24 | 3.09 | 0.17 | 3.19 | 0.10 | 3.20 | 0.10 | 3.49 | 0.09 |
|  | Low-budget restaurants | 4.26 | 0.15 | 3.84 | 0.19 | 4.37 | 0.31 | 3.61 | 0.17 | 4.61 | 0.13 | 5.35 | 0.14 | 3.79 | 0.11 |
|  | Public markets | 9.86 | 0.38 | 10.44 | 0.43 | 9.48 | 0.70 | 8.93 | 0.47 | 8.75 | 0.27 | 7.85 | 0.24 | 7.86 | 0.22 |
|  | Specialty stores & small neighborhood stores | 61.65 | 0.50 | 59.43 | 0.58 | 58.36 | 0.95 | 59.84 | 0.68 | 60.49 | 0.37 | 60.64 | 0.35 | 63.17 | 0.32 |
|  | Convenience stores | 0.41 | 0.05 | 0.38 | 0.05 | 0.63 | 0.09 | 0.65 | 0.07 | 0.59 | 0.03 | 0.81 | 0.04 | 0.83 | 0.04 |
|  | Supermarkets & Department stores | 6.97 | 0.28 | 7.11 | 0.31 | 8.18 | 0.55 | 7.70 | 0.38 | 7.13 | 0.19 | 7.06 | 0.19 | 6.50 | 0.16 |
|  | Restaurants, cafes, bars | 1.01 | 0.07 | 1.38 | 0.09 | 1.31 | 0.15 | 1.25 | 0.11 | 1.24 | 0.06 | 1.27 | 0.06 | 0.68 | 0.04 |
|  | Other stores | 1.88 | 0.16 | 2.41 | 0.30 | 2.53 | 0.48 | 2.48 | 0.27 | 1.79 | 0.12 | 1.88 | 0.12 | 1.58 | 0.10 |
| High School | Street markets & street vendors | 10.97 | 0.25 | 10.24 | 0.29 | 10.04 | 0.46 | 10.29 | 0.30 | 10.38 | 0.18 | 10.10 | 0.17 | 10.19 | 0.17 |
|  | Acquaintances | - | - | 2.26 | 0.11 | 3.01 | 0.31 | 2.05 | 0.11 | 2.40 | 0.07 | 2.33 | 0.07 | 2.62 | 0.06 |
|  | Low-budget restaurants | 7.52 | 0.23 | 6.36 | 0.22 | 6.95 | 0.42 | 6.04 | 0.24 | 6.85 | 0.15 | 7.59 | 0.15 | 5.33 | 0.12 |
|  | Public markets | 10.04 | 0.32 | 10.29 | 0.32 | 9.67 | 0.64 | 8.75 | 0.38 | 8.74 | 0.26 | 8.25 | 0.24 | 8.67 | 0.22 |
|  | Specialty stores & small neighborhood stores | 52.72 | 0.51 | 53.11 | 0.51 | 52.10 | 0.94 | 52.85 | 0.62 | 53.58 | 0.34 | 54.78 | 0.35 | 58.43 | 0.30 |
|  | Convenience stores | 0.80 | 0.08 | 1.01 | 0.10 | 1.07 | 0.11 | 1.34 | 0.10 | 1.35 | 0.05 | 1.39 | 0.05 | 1.42 | 0.04 |
|  | Supermarkets & Department stores | 12.41 | 0.34 | 12.40 | 0.37 | 12.72 | 0.70 | 13.61 | 0.45 | 11.95 | 0.22 | 11.26 | 0.22 | 10.70 | 0.18 |
|  | Restaurants, cafes, bars | 3.48 | 0.20 | 3.53 | 0.17 | 3.53 | 0.30 | 3.93 | 0.20 | 3.77 | 0.12 | 3.45 | 0.10 | 1.75 | 0.07 |
|  | Other stores | 2.06 | 0.13 | 0.82 | 0.09 | 0.90 | 0.12 | 1.15 | 0.12 | 0.98 | 0.05 | 0.85 | 0.04 | 0.88 | 0.04 |
| Higher education | Street markets & street vendors | 7.89 | 0.33 | 6.50 | 0.29 | 7.12 | 0.62 | 6.57 | 0.34 | 7.09 | 0.26 | 7.09 | 0.25 | 7.73 | 0.25 |
|  | Acquaintances | - | - | 1.94 | 0.19 | 2.05 | 0.41 | 1.34 | 0.14 | 1.62 | 0.09 | 1.62 | 0.09 | 2.14 | 0.10 |
|  | Low-budget restaurants | 9.90 | 0.42 | 8.14 | 0.43 | 10.02 | 0.87 | 10.05 | 0.64 | 9.84 | 0.34 | 11.53 | 0.34 | 6.49 | 0.24 |
|  | Public markets | 9.16 | 0.49 | 8.35 | 0.43 | 8.59 | 0.98 | 7.99 | 0.56 | 8.40 | 0.35 | 8.14 | 0.34 | 8.75 | 0.34 |
|  | Specialty stores & small neighborhood stores | 33.17 | 0.81 | 33.97 | 0.85 | 31.66 | 1.41 | 34.12 | 0.97 | 34.20 | 0.55 | 36.46 | 0.58 | 44.29 | 0.53 |
|  | Convenience stores | 1.91 | 0.28 | 2.41 | 0.35 | 2.18 | 0.41 | 2.34 | 0.21 | 2.26 | 0.13 | 2.22 | 0.10 | 2.49 | 0.11 |
|  | Supermarkets & Department stores | 22.49 | 0.76 | 22.33 | 0.85 | 22.17 | 1.47 | 22.76 | 0.91 | 20.05 | 0.51 | 18.23 | 0.42 | 19.55 | 0.44 |
|  | Restaurants, cafes, bars | 12.56 | 0.61 | 15.24 | 0.75 | 14.50 | 1.27 | 13.73 | 0.71 | 15.14 | 0.45 | 13.56 | 0.45 | 7.05 | 0.31 |
|  | Other stores | 2.92 | 0.30 | 1.13 | 0.15 | 1.70 | 0.33 | 1.10 | 0.15 | 1.39 | 0.12 | 1.14 | 0.09 | 1.52 | 0.12 |

Chain convenience stores start being considered by ENIGH in 2006 and Acquaintances in 2010

**Supplementary Table 5.** Food and beverage purchases (% expenditure) by store type stratified by urbanicity, ENIGH 1994 – 2020.

| Urbanicity | Food Outlets | 1994 | | 1996 | | 1998 | | 2000 | | 2002 | | 2004 | | 2006 | |
| --- | --- | --- | --- | --- | --- | --- | --- | --- | --- | --- | --- | --- | --- | --- | --- |
|  |  | % | SE | % | SE | % | SE | % | SE | % | SE | % | SE | % | SE |
| Rural localities | Street markets & street vendors | 19.90 | 1.24 | 16.10 | 0.88 | 16.90 | 1.38 | 18.80 | 1.23 | 21.30 | 2.18 | 16.80 | 1.54 | 17.70 | 0.79 |
|  | Acquaintances | - | - | - | - | - | - | - | - | - | - | - | - | - | - |
|  | Low-budget restaurants | 1.50 | 0.30 | 1.50 | 0.21 | 1.00 | 0.15 | 1.30 | 0.18 | 2.80 | 0.44 | 4.00 | 0.50 | 4.70 | 0.34 |
|  | Public markets | 7.90 | 0.83 | 5.60 | 0.60 | 5.20 | 0.84 | 7.30 | 1.14 | 4.90 | 0.69 | 6.50 | 1.15 | 10.60 | 2.67 |
|  | Specialty stores & small neighborhood stores | 65.80 | 1.51 | 69.65 | 1.28 | 72.20 | 1.56 | 67.60 | 1.40 | 65.10 | 2.07 | 66.20 | 1.74 | 59.40 | 2.26 |
|  | Convenience stores | - | - | - | - | - | - | - | - | - | - | - | - | 0.10 | 0.01 |
|  | Supermarkets & department stores | 2.80 | 0.44 | 3.20 | 0.63 | 2.30 | 0.42 | 2.50 | 0.55 | 2.70 | 0.58 | 2.60 | 0.40 | 3.00 | 0.44 |
|  | Restaurants, cafes, bars | 0.40 | 0.08 | 0.40 | 0.11 | 0.30 | 0.07 | 0.60 | 0.14 | 1.40 | 0.85 | 1.40 | 0.20 | 1.40 | 0.17 |
|  | Other stores | 1.70 | 0.24 | 3.60 | 0.46 | 2.00 | 0.29 | 1.90 | 0.32 | 1.80 | 0.31 | 2.70 | 0.45 | 3.20 | 0.32 |
| Small cities | Street markets & street vendors | 12.40 | 1.12 | 14.40 | 1.48 | 12.00 | 1.35 | 13.30 | 1.68 | 14.70 | 1.46 | 9.90 | 1.05 | 13.20 | 0.90 |
|  | Acquaintances | - | - | - | - | - | - | - | - | - | - | - | - | - | - |
|  | Low-budget restaurants | 3.40 | 0.87 | 2.80 | 0.58 | 2.10 | 0.56 | 3.30 | 0.75 | 4.00 | 0.46 | 8.40 | 1.00 | 6.30 | 0.77 |
|  | Public markets | 15.10 | 3.34 | 11.50 | 1.58 | 13.90 | 3.08 | 12.00 | 2.75 | 12.60 | 1.86 | 8.70 | 1.50 | 10.60 | 1.97 |
|  | Specialty stores & small neighborhood stores | 61.00 | 3.08 | 66.70 | 2.07 | 67.00 | 2.83 | 63.80 | 2.65 | 59.00 | 3.49 | 66.70 | 2.29 | 62.70 | 1.97 |
|  | Convenience stores | - | - | - | - | - | - | - | - | - | - | - | - | 0.00 | 0.01 |
|  | Supermarkets & department stores | 4.50 | 1.31 | 2.40 | 0.50 | 2.50 | 0.72 | 3.90 | 1.64 | 6.40 | 2.38 | 3.00 | 0.70 | 2.60 | 0.43 |
|  | Restaurants, cafes, bars | 1.80 | 0.47 | 0.80 | 0.21 | 0.50 | 0.13 | 1.90 | 0.54 | 1.80 | 0.41 | 2.00 | 0.53 | 1.20 | 0.24 |
|  | Other stores | 1.70 | 0.93 | 1.40 | 0.26 | 2.00 | 0.39 | 1.80 | 0.35 | 1.40 | 0.38 | 1.30 | 0.35 | 3.40 | 0.45 |
| Medium cities | Street markets & street vendors | 10.70 | 1.13 | 11.10 | 0.79 | 11.10 | 1.25 | 9.40 | 1.07 | 11.10 | 1.01 | 8.40 | 0.56 | 10.20 | 0.55 |
|  | Acquaintances | - | - | - | - | - | - | - | - | - | - | - | - | - | - |
|  | Low-budget restaurants | 4.40 | 0.46 | 2.30 | 0.29 | 2.20 | 0.33 | 3.00 | 0.46 | 4.00 | 0.33 | 6.90 | 0.45 | 6.80 | 0.41 |
|  | Public markets | 21.50 | 3.34 | 16.20 | 1.96 | 16.60 | 2.36 | 15.20 | 2.10 | 12.20 | 1.33 | 11.50 | 1.21 | 12.00 | 1.36 |
|  | Specialty stores & small neighborhood stores | 51.80 | 3.03 | 60.60 | 1.97 | 59.10 | 2.36 | 60.50 | 2.09 | 58.30 | 1.75 | 60.30 | 1.25 | 54.80 | 1.12 |
|  | Convenience stores | - | - | - | - | - | - | - | - | - | - | - | - | 0.30 | 0.08 |
|  | Supermarkets & department stores | 8.00 | 1.21 | 7.40 | 1.19 | 8.60 | 1.42 | 8.50 | 1.42 | 9.30 | 1.12 | 7.00 | 0.72 | 9.30 | 0.77 |
|  | Restaurants, cafes, bars | 1.60 | 0.35 | 1.20 | 0.27 | 1.10 | 0.19 | 2.20 | 0.50 | 3.20 | 0.57 | 3.90 | 0.41 | 3.50 | 0.25 |
|  | Other stores | 2.00 | 0.49 | 1.10 | 0.20 | 1.40 | 0.29 | 1.20 | 0.27 | 2.00 | 0.39 | 2.00 | 0.28 | 3.00 | 0.32 |
| Metropolitan cities | Street markets & street vendors | 9.20 | 0.51 | 10.10 | 0.53 | 9.30 | 0.34 | 9.80 | 0.55 | 9.40 | 0.45 | 7.60 | 0.19 | 10.60 | 0.25 |
|  | Acquaintances | - | - | - | - | - | - | - | - | - | - | - | - | - | - |
|  | Low-budget restaurants | 4.80 | 0.20 | 3.20 | 0.25 | 3.60 | 0.25 | 5.40 | 0.48 | 5.70 | 0.32 | 8.10 | 0.23 | 8.80 | 0.24 |
|  | Public markets | 18.20 | 1.56 | 18.00 | 1.21 | 16.30 | 0.79 | 12.50 | 1.12 | 14.00 | 0.84 | 11.30 | 0.32 | 10.70 | 0.37 |
|  | Specialty stores & small neighborhood stores | 46.50 | 1.53 | 47.60 | 1.29 | 50.60 | 0.86 | 49.60 | 1.50 | 49.50 | 1.00 | 50.60 | 0.50 | 45.40 | 0.51 |
|  | Convenience stores | - | - | - | - | - | - | - | - | - | - | - | - | 0.80 | 0.06 |
|  | Supermarkets & department stores | 14.60 | 1.31 | 16.70 | 1.49 | 16.10 | 0.72 | 16.70 | 1.55 | 15.10 | 0.64 | 14.30 | 0.36 | 14.70 | 0.34 |
|  | Restaurants, cafes, bars | 5.40 | 1.02 | 3.00 | 0.34 | 2.60 | 0.23 | 4.10 | 0.45 | 5.10 | 0.36 | 6.60 | 0.23 | 6.20 | 0.24 |
|  | Other stores | 1.30 | 0.22 | 1.20 | 0.16 | 1.50 | 0.16 | 1.80 | 0.24 | 1.20 | 0.12 | 1.50 | 0.09 | 2.80 | 0.15 |

**Supplementary Table 5.** Continuation

| Urbanicity | Food Outlets | 2008 | | 2010 | | 2012 | | 2014 | | 2016 | | 2018 | | 2020 | |
| --- | --- | --- | --- | --- | --- | --- | --- | --- | --- | --- | --- | --- | --- | --- | --- |
|  |  | % | SE | % | SE | % | SE | % | SE | % | SE | % | SE | % | SE |
| Rural localities | Street markets & street vendors | 18.50 | 0.88 | 15.40 | 0.92 | 13.80 | 0.76 | 15.40 | 0.71 | 14.10 | 0.37 | 14.10 | 0.34 | 14.00 | 0.33 |
|  | Acquaintances | - | - | 5.00 | 0.41 | 5.80 | 0.56 | 4.60 | 0.31 | 5.20 | 0.20 | 5.00 | 0.17 | 5.50 | 0.15 |
|  | Low-budget restaurants | 3.70 | 0.25 | 2.80 | 0.24 | 3.40 | 0.30 | 3.50 | 0.25 | 4.10 | 0.15 | 4.50 | 0.13 | 3.60 | 0.11 |
|  | Public markets | 6.10 | 0.56 | 7.30 | 0.66 | 6.40 | 0.94 | 5.50 | 0.54 | 5.60 | 0.31 | 5.00 | 0.25 | 4.60 | 0.24 |
|  | Specialty stores & small neighborhood stores | 66.10 | 1.04 | 59.00 | 1.29 | 59.20 | 1.55 | 58.60 | 1.18 | 61.40 | 0.54 | 62.00 | 0.49 | 64.10 | 0.44 |
|  | Convenience stores | 0.10 | 0.01 | 0.10 | 0.08 | 0.10 | 0.03 | 0.40 | 0.16 | 0.20 | 0.02 | 0.20 | 0.02 | 0.30 | 0.02 |
|  | Supermarkets & department stores | 2.50 | 0.29 | 2.90 | 0.40 | 2.90 | 0.47 | 4.50 | 0.49 | 3.80 | 0.18 | 3.80 | 0.18 | 3.60 | 0.14 |
|  | Restaurants, cafes, bars | 0.60 | 0.07 | 1.10 | 0.16 | 1.30 | 0.21 | 1.30 | 0.19 | 1.20 | 0.09 | 1.10 | 0.06 | 0.70 | 0.04 |
|  | Other stores | 2.50 | 0.21 | 6.30 | 0.87 | 7.10 | 1.35 | 6.30 | 0.71 | 4.30 | 0.30 | 4.40 | 0.28 | 3.70 | 0.23 |
| Small cities | Street markets & street vendors | 13.40 | 0.81 | 12.70 | 1.16 | 12.10 | 1.10 | 12.20 | 0.82 | 11.50 | 0.48 | 10.70 | 0.40 | 10.80 | 0.49 |
|  | Acquaintances | - | - | 2.40 | 0.20 | 2.80 | 0.42 | 3.20 | 0.32 | 3.20 | 0.19 | 3.50 | 0.25 | 3.70 | 0.19 |
|  | Low-budget restaurants | 4.70 | 0.39 | 4.60 | 0.58 | 5.90 | 0.55 | 4.90 | 0.38 | 5.50 | 0.27 | 6.40 | 0.26 | 4.80 | 0.22 |
|  | Public markets | 11.40 | 1.21 | 11.30 | 1.15 | 11.10 | 2.07 | 8.20 | 0.99 | 7.40 | 0.70 | 7.30 | 0.62 | 6.20 | 0.43 |
|  | Specialty stores & small neighborhood stores | 64.30 | 1.55 | 61.60 | 1.53 | 62.30 | 2.23 | 64.00 | 1.35 | 64.10 | 0.87 | 64.20 | 0.78 | 67.90 | 0.64 |
|  | Convenience stores | 0.10 | 0.04 | 0.20 | 0.06 | 0.40 | 0.16 | 0.20 | 0.07 | 0.40 | 0.06 | 0.40 | 0.05 | 0.60 | 0.06 |
|  | Supermarkets & department stores | 2.50 | 0.33 | 4.10 | 1.06 | 3.20 | 0.69 | 4.50 | 0.62 | 5.10 | 0.44 | 4.80 | 0.41 | 4.30 | 0.34 |
|  | Restaurants, cafes, bars | 1.60 | 0.34 | 1.60 | 0.21 | 1.40 | 0.40 | 1.60 | 0.20 | 1.90 | 0.18 | 2.10 | 0.15 | 1.10 | 0.09 |
|  | Other stores | 2.00 | 0.33 | 1.50 | 0.52 | 0.80 | 0.22 | 1.20 | 0.35 | 0.90 | 0.13 | 0.60 | 0.07 | 0.80 | 0.12 |
| Medium cities | Street markets & street vendors | 9.70 | 0.40 | 8.40 | 0.36 | 7.70 | 0.63 | 8.40 | 0.53 | 8.80 | 0.36 | 8.10 | 0.39 | 8.50 | 0.36 |
|  | Acquaintances | - | - | 2.30 | 0.20 | 3.00 | 0.47 | 2.20 | 0.24 | 2.30 | 0.14 | 2.30 | 0.14 | 2.50 | 0.12 |
|  | Low-budget restaurants | 6.60 | 0.33 | 5.70 | 0.37 | 6.50 | 0.75 | 5.30 | 0.43 | 6.30 | 0.30 | 7.40 | 0.31 | 5.10 | 0.22 |
|  | Public markets | 10.80 | 0.83 | 10.50 | 0.81 | 11.20 | 1.57 | 9.40 | 0.88 | 11.00 | 0.70 | 10.70 | 0.63 | 9.40 | 0.50 |
|  | Specialty stores & small neighborhood stores | 58.50 | 0.87 | 59.60 | 0.97 | 58.60 | 1.97 | 57.90 | 1.34 | 56.20 | 0.80 | 56.50 | 0.80 | 61.40 | 0.66 |
|  | Convenience stores | 0.60 | 0.13 | 0.40 | 0.08 | 0.70 | 0.25 | 0.80 | 0.17 | 1.10 | 0.13 | 1.20 | 0.12 | 1.20 | 0.09 |
|  | Supermarkets & department stores | 9.50 | 0.68 | 10.00 | 0.64 | 8.90 | 1.20 | 12.40 | 1.14 | 10.60 | 0.54 | 9.90 | 0.55 | 9.80 | 0.45 |
|  | Restaurants, cafes, bars | 2.30 | 0.19 | 3.00 | 0.27 | 3.10 | 0.80 | 3.10 | 0.34 | 3.20 | 0.21 | 3.40 | 0.23 | 1.70 | 0.13 |
|  | Other stores | 2.00 | 0.19 | 0.20 | 0.04 | 0.30 | 0.12 | 0.40 | 0.11 | 0.50 | 0.08 | 0.40 | 0.05 | 0.40 | 0.04 |
| Metropolitan cities | Street markets & street vendors | 10.40 | 0.19 | 9.40 | 0.20 | 10.20 | 0.49 | 9.50 | 0.31 | 10.00 | 0.19 | 9.60 | 0.19 | 9.90 | 0.19 |
|  | Acquaintances | - | - | 1.70 | 0.09 | 2.20 | 0.25 | 1.40 | 0.09 | 1.60 | 0.06 | 1.40 | 0.05 | 1.90 | 0.05 |
|  | Low-budget restaurants | 7.00 | 0.18 | 6.20 | 0.19 | 6.90 | 0.44 | 6.40 | 0.26 | 7.20 | 0.16 | 8.40 | 0.17 | 5.30 | 0.13 |
|  | Public markets | 10.80 | 0.28 | 10.90 | 0.30 | 9.60 | 0.66 | 10.00 | 0.49 | 9.70 | 0.27 | 9.00 | 0.27 | 10.30 | 0.28 |
|  | Specialty stores & small neighborhood stores | 48.00 | 0.43 | 48.50 | 0.48 | 46.20 | 1.03 | 47.70 | 0.64 | 47.60 | 0.37 | 48.00 | 0.39 | 52.40 | 0.34 |
|  | Convenience stores | 1.20 | 0.09 | 1.40 | 0.14 | 1.50 | 0.14 | 1.80 | 0.10 | 1.70 | 0.06 | 2.00 | 0.06 | 2.00 | 0.06 |
|  | Supermarkets & department stores | 15.90 | 0.34 | 15.70 | 0.38 | 17.40 | 0.80 | 16.80 | 0.48 | 15.30 | 0.25 | 15.10 | 0.24 | 14.50 | 0.22 |
|  | Restaurants, cafes, bars | 4.80 | 0.20 | 5.70 | 0.23 | 5.40 | 0.39 | 5.90 | 0.29 | 6.30 | 0.18 | 6.00 | 0.19 | 3.00 | 0.11 |
|  | Other stores | 2.00 | 0.13 | 0.50 | 0.05 | 0.60 | 0.10 | 0.50 | 0.05 | 0.60 | 0.04 | 0.60 | 0.04 | 0.70 | 0.04 |

Chain convenience stores start being considered by ENIGH in 2006 and Acquaintances in 2010
